# The Xbp1-regulated transcription factor Mist1 restricts antibody secretion by restraining Blimp1 expression in plasma cells

**DOI:** 10.3389/fimmu.2022.859598

**Published:** 2022-12-21

**Authors:** Miriam Wöhner, Theresa Pinter, Peter Bönelt, Astrid Hagelkruys, Daniela Kostanova-Poliakova, Johannes Stadlmann, Stephen F. Konieczny, Maria Fischer, Markus Jaritz, Meinrad Busslinger

**Affiliations:** ^1^ Research Institute of Molecular Pathology (IMP), Vienna Biocenter (VBC), Vienna, Austria; ^2^ Institute of Molecular Biotechnology of the Austrian Academy of Sciences (IMBA), Vienna Biocenter (VBC), Vienna, Austria; ^3^ Department of Biological Science, Purdue University, West Lafayette, IN, United States

**Keywords:** Mist1 (Bhlha15), Blimp1 (Prdm1), XBP1, plasma cell differentiation, antibody secretion, unfolded protein response (UPR), gene regulation

## Abstract

Antibody secretion by plasma cells provides acute and long-term protection against pathogens. The high secretion potential of plasma cells depends on the unfolded protein response, which is controlled by the transcription factor Xbp1. Here, we analyzed the Xbp1-dependent gene expression program of plasma cells and identified *Bhlha15* (Mist1) as the most strongly activated Xbp1 target gene. As Mist1 plays an important role in other secretory cell types, we analyzed in detail the phenotype of Mist1-deficient plasma cells in *Cd23*-Cre *Bhlha15* ^fl/fl^ mice under steady-state condition or upon NP-KLH immunization. Under both conditions, Mist1-deficient plasma cells were 1.4-fold reduced in number and exhibited increased IgM production and antibody secretion compared to control plasma cells. At the molecular level, Mist1 regulated a largely different set of target genes compared with Xbp1. Notably, expression of the Blimp1 protein, which is known to activate immunoglobulin gene expression and to contribute to antibody secretion, was 1.3-fold upregulated in Mist1-deficient plasma cells, which led to a moderate downregulation of most Blimp1-repressed target genes in the absence of Mist1. Importantly, a 2-fold reduction of Blimp1 (*Prdm1*) expression was sufficient to restore the cell number and antibody expression of plasma cells in *Prdm1* ^Gfp/+^ *Cd23*-Cre *Bhlha15* ^fl/fl^ mice to the same level seen in control mice. Together, these data indicate that Mist1 restricts antibody secretion by restraining Blimp1 expression, which likely contributes to the viability of plasma cells.

## Introduction

Plasma cells (PCs) provide protection of the host against infection by secreting high-affinity antibodies that recognize an almost unlimited number of pathogens (1). Activation of mature B cells in peripheral lymphoid organs leads to the differentiation of short-lived, antibody-secreting plasmablasts (PBs) and PCs, which can subsequently develop into quiescent long-lived PCs upon migration to survival niches in the bone marrow (1). The differentiation of mature B cells to PBs is associated with substantial changes in gene expression and cell morphology (1, 2). While the expression of B cell-specific regulators, like Pax5 and Ebf1, which maintain the B cell gene expression program, is downregulated during PB formation (2, 3), the increased expression of Irf4 and Blimp1 (*Prdm1*) promotes PB differentiation by activating PC-specific genes and repressing the B cell gene expression program (4–6). During the process of PB differentiation, the rearranged antigen receptor loci encoding the immunoglobulin heavy chain (*Igh*) and light chain (*Igk* and *Igl*) genes are strongly activated, and the *Igh* gene transcripts furthermore undergo a posttranscriptional expression switch from the membrane-bound form to the secreted form of the Ig heavy chain, which results in the production and secretion of large amounts of antibodies (1, 3). The PBs and PCs thereby undergo a massive change in morphology by enlarging their endoplasmic reticulum (ER), which promotes efficient antibody secretion and ensures their cell survival (1, 7).

The morphological change of PBs and PCs is a direct consequence of the unfolded protein response (UPR), which is induced by protein overloading of the ER and restores the folding, processing and export of proteins that pass through the ER (8). The essential UPR pathway of PBs and PCs is under the control of the ER-resident transmembrane protein Ire1α (*Ern1*), which is normally inactivated by the inhibitory Hsp70-type chaperone BiP (*Hsp5*) inside of the ER (8). Upon accumulation of unfolded proteins in the ER, BiP dissociates from Ire1α, which in turn activates the endoribonuclease activity of Ire1α that splices out 26 nucleotides from the cytoplasmic *Xbp1* mRNA, thus leading to a frameshift mediating translation of the transcriptionally active regulator Xbp1s (9, 10). Xbp1s activates the UPR gene expression program, thus resulting in a massive expansion of the ER and secretory protein apparatus (7). Consequently, antibody secretion is severely impaired upon inactivation of *Xbp1* in PBs and PCs (11–13). Xbp1 is, however, not essential for the generation of PBs and PCs (12), and its gene-regulatory function in these antibody-secreting cells has not yet been fully explored.

Our investigation of the Xbp1-dependent gene expression program in PBs by ChIP- and RNA-seq analyses revealed that the most strongly activated Xbp1 target gene is *Bhlha15* coding for the transcription factor Mist1 (14). Mist1 was previously shown to play an important role in other secretory cell types by inducing and maintaining their secretory cell architecture (14–18). Here, we used conditional gene inactivation combined with RNA- and ChIP-seq analyses to systematically investigate the role of Mist1 in PCs. Mist1-deficient PCs were decreased in the spleen and bone marrow under steady-state condition and upon immunization. Moreover, the antibody secretion was increased in Mist1-deficient PCs, in marked contrast to Xbp1-deficient PCs, as previously shown (12, 13). At the molecular level, Xbp1 and Mist1 regulated a largely different set of target genes. Notably, the PC-specific regulator Blimp1 was upregulated in Mist1-deficient PCs, which resulted in a moderate downregulation of most Blimp1-repressed target genes. Importantly, a two-fold reduction of Blimp1 expression in Mist1-deficient PCs rescued PC numbers and reduced antibody secretion to the same level observed in control PCs. Hence, these data indicate that Mist1 largely mediates its effects on plasma cells by restricting antibody secretion through restraining Blimp1 expression, which likely imparts PC viability.

## Results

### Analysis of the Xbp1-regulated gene expression program in plasmablasts

To identify regulated Xbp1 target genes contributing to antibody secretion in PBs and PCs, we performed RNA-seq and ChIP-seq analyses with *in vitro* generated PBs. To this end, we cultured splenic CD43^–^ B cells for 8 days in the iGB system on 40LB feeder cells (19). In this system, which mimics T cell help, naïve B cells are initially stimulated with BAFF, CD40 ligand and IL-4 for 4 days, followed by 4 days of stimulation with BAFF, CD40 ligand and IL-21, which promotes PB differentiation (19).

**Figure 1:**
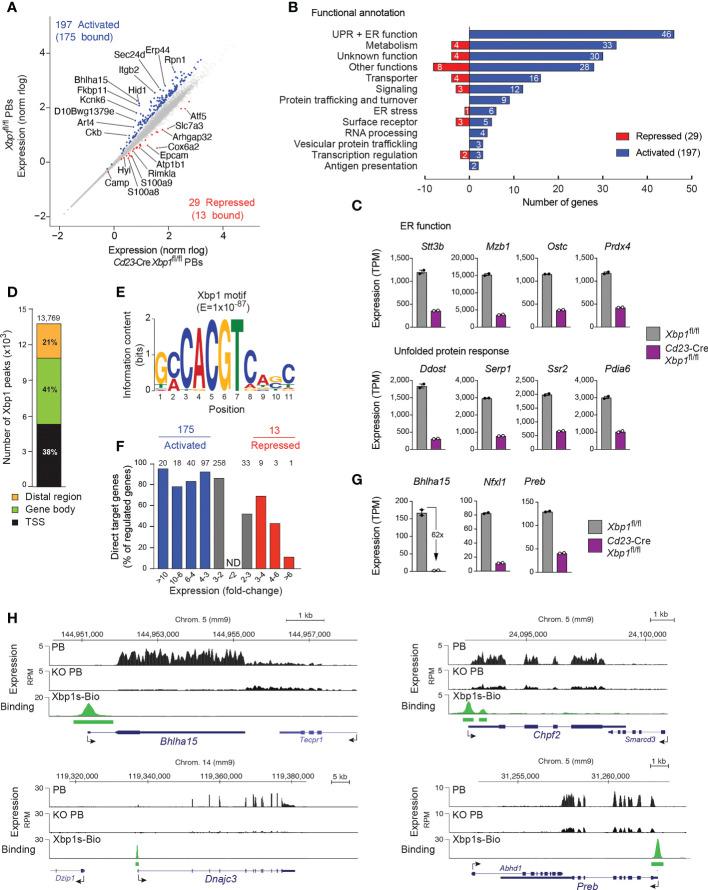
Xbp1-dependent gene expression program in plasmablasts. **(A)** Scatter plot of gene expression differences between *Cd23*-Cre *Xbp1* ^fl/fl^ and *Xbp1* ^fl/fl^ PBs that were sorted as CD19^+^CD138^+^CD23^–^cells after stimulation in the iGB system for 4 days with IL-4 followed by 4 days with IL-21. Two independent RNA-seq experiments were performed with PBs of each genotype. Each dot corresponds to one gene, whose expression is plotted as normalized log_10_ (norm rlog) expression value. Genes with an expression difference of > 3-fold, an adjusted *P* value of < 0.05, a transcripts per million (TPM) expression value of > 5 (at least in one sample) are colored in blue or red corresponding to activation or repression by Xbp1, respectively. **(B)** Functional classification and quantification (number) of proteins encoded by Xbp1-activated and Xbp1-repressed genes. **(C)** Expression of genes involved in UPR or ER function, shown as mean TPM values of two RNA-seq experiments of *Cd23*-Cre *Xbp1* ^fl/fl^ or *Xbp1* ^fl/fl^ PBs, respectively. **(D)** Presence of Xbp1 peaks in distal regions, gene bodies and promoter (TSS) regions. Splenic B cells from *Xbp1* ^Bio/Bio^ *Rosa26* ^BirA/+^ mice were stimulated for 4 days with LPS followed by Bio-ChIP-seq analysis. The Xbp1 peaks were assigned to genes as described (22). **(E)** Consensus Xbp1-binding motif identified with an E-value of 1 x 10^-87^ by the *de novo* motif-discovery program MEME-Chip. **(F)** The number of regulated target genes is shown for the indicated fold-change of gene expression between *Cd23*-Cre *Xbp1* ^fl/fl^ and *Xbp1* ^fl/fl^ PBs. Grey bars indicated activated or repressed genes with less than a 3-fold expression change. **(G)** Expression of Xbp1-activated transcription factor genes in *Cd23*-Cre *Xbp1* ^fl/fl^ and *Xbp1* ^fl/fl^ PBs, shown as mean TPM values of two RNA-seq experiments per genotype. **(H)** Xbp1 binding and RNA-seq expression at selected activated Xbp1 target loci. Horizontal bars indicate Xbp1s-Bio peaks identified by MACS peak calling.

For conditional inactivation of the floxed (fl) *Xbp1* allele (20), we used the *Cd23*-Cre line, which initiates Cre-mediated deletion in immature B cells and leads to efficient deletion in mature B cells of the spleen (21). B cells from the spleen of *Cd23*-Cre *Xbp1* ^fl/fl^ and control *Xbp1* ^fl/fl^ mice were differentiated in the iGB system to PBs, which were sorted as CD19^+^CD138^+^CD23^–^ cells for RNA-seq analysis (**Supplementary Figures 1A, B**). We identified 197 Xbp1-activated and 29 Xbp1-repressed genes, which were selected for an expression difference of > 3-fold, an adjusted *P* value of < 0.05 and an expression value of > 5 transcripts per million (TPM) in *Xbp1* ^fl/fl^ PBs (activated genes) or *Cd23*-Cre *Xbp1* ^fl/fl^ PBs (repressed genes), respectively (**Figure 1A** and **Supplementary Table 1**). The 197 Xbp1-activated genes were annotated and grouped according to their function. The two largest functional groups consisted of 46 genes involved in UPR or ER function and 33 genes implicated in metabolism (**Figures 1B, C**), which is consistent with a critical role of Xbp1 in orchestrating antibody synthesis, modification and secretion as well as metabolic reprogramming of PBs (7). We next compared the identified Xbp1-activated genes by gene set enrichment analysis (GSEA) with a published dataset obtained with *ex vivo* sorted Xbp1-deficient plasma cells (13). Notably, the Xbp1-activated genes, which were downregulated upon loss of Xbp1 in *in vivo* plasma cells, were also downregulated in our dataset (**Supplementary Figure 1C**). Moreover, we also observed a strong correlation of expression between known UPR genes (13) and the Xbp1-regulated genes of our dataset (**Supplementary Figure 1C**), in agreement with the fact that many UPR genes are regulated by Xbp1 (8).

As the development and function of PBs and PCs is critically dependent on the transcription factors Irf4 (23), Blimp1 (*Prdm1*) (24), Ikaros (*Ikzf1*) (25), Aiolos (*Ikzf3*) (26), E2A (*Tcf3*) and E2-2 (*Tcf4*) (27), we next analyzed the expression of these genes in Xbp1-deficient PBs. All 6 genes were expressed at a moderately elevated level in the *Cd23*-Cre *Xbp1* ^fl/fl^ PBs compared with control *Xbp1* ^fl/fl^ PBs (**Supplementary Figure 1D**). *Pax5*, which is downregulated in the transition from mature B cells to PCs (28), was expressed at an equally low level in Xbp1-deficient and control PBs. Hence, Xbp1 does not play a crucial role in the regulation of key transcription factors that control plasma cell development and function.

To be able to analyze the genome-wide pattern of Xbp1 binding by ChIP-seq, we generated a *Xbp1s* ^Bio^ allele by inserting a biotin acceptor sequence at the last codon of *Xbp1s* (**Supplementary Figure 1E**). To facilitate *in vivo* biotinylation of the biotin acceptor sequence by the *E.coli* BirA ligase, we generated *Xbp1s* ^Bio/Bio^ *Rosa26* ^BirA/BirA^ mice, which exhibited normal B cell development and only a small increase in bone marrow PCs (**Supplementary Figure 1F**). Importantly, the Mist1 protein, encoded by an activated Xbp1 target gene (see below), was similarly expressed in PCs from the bone marrow of *Xbp1s* ^Bio/Bio^ *Rosa26* ^BirA/BirA^ and control *Rosa26* ^BirA/BirA^ mice, indicating that the C-terminal addition of the biotin acceptor sequence did not interfere with the transcriptional activity of Xbp1s-Bio (**Supplementary Figure 1G**). Moreover, enzyme-linked immunospot (ELISPOT) assays furthermore revealed similar numbers of IgM and IgG antibody-secreting cells (ASC) in the bone marrow, indicating that the plasma cells of *Xbp1s* ^Bio/Bio^ *Rosa26* ^BirA/BirA^ mice were functional (**Supplementary Figure 1H**). We next stimulated splenic *Xbp1s* ^Bio/Bio^ *Rosa26* ^BirA/+^ B cells with lipopolysaccharide (LPS) for 4 days and performed streptavidin-mediated pulldown of the Xbp1s-Bio protein from nuclear extracts of these LPS-stimulated cells followed by Bio-ChIP-seq analysis (22). Peak calling with a stringent *P* value of < 10^–10^ identified 13,769 Xbp1 peaks, which were primarily located in promoter regions (38%) and gene bodies (41%) (**Figure 1D**). Analysis of the Xbp1 peak sequences with a *de novo* motif discovery program identified a consensus Xbp1-binding motif (**Figure 1E**), which resembles a published Xbp1 recognition sequence (29) (**Supplementary Figure 1l**). Peak-to-gene assignment defined 9,210 Xbp1-bound genes. By determining the overlap between these Xbp1-bound genes and the Xbp1-regulated genes (**Figure 1A**), we identified 175 potentially directly activated Xbp1 target genes (corresponding to 89% of all activated genes) and 13 potentially directly repressed target genes (44%, **Figures 1A, F** and **Supplementary Table 1**). Consistent with this finding, gene activation clearly correlated with Xbp1 binding in marked contrast to the inverse correlation of Xbp1 binding with increasing gene repression (**Figure 1F**). These data strongly suggest that Xbp1s is a dedicated transcriptional activator.

Xbp1 directly activated the 3 transcription factor genes *Bhlha15*, *Nfxl1* and *Preb* in PBs (**Figures 1G, H**). Other examples of directly and strongly regulated Xbp1 targets include the metabolic gene *Chpf2* (30) and UPR gene *Dnajc3* (31) (**Figure 1H**). Notably, *Bhlha15* was 62-fold activated by Xbp1 in PBs and was thus the most strongly activated Xbp1 target gene, showing prominent Xbp1 binding at its promoter (**Figures 1G, H**). This finding is consistent with previous reports, demonstrating that Xbp1 binds to and regulates *Bhlha15* in gastric cells and fibroblasts (16, 32). The *Bhlha15* gene, which codes for the transcription factor Mist1 (14), was strongly activated during *in vitro* differentiation in the iGB system from the activated B cell stage to pre-PBs and PBs as well as *in vivo* during the developmental transition from mature and germinal center (GC) B cells to plasma cells (**Supplementary Figure 2A**). Despite its prominent PC-specific expression pattern, Mist1 was previously reported to play only a minimal role in *in vitro* differentiated PBs (33) and *in vivo* splenic PCs (34). We therefore decided to reinvestigate in a systematic manner the function of Mist1 *in vivo* in PCs under steady-state conditions and upon immunization.

### Moderate plasma cell reduction and increased antibody secretion upon loss of Mist1

**Figure 2:**
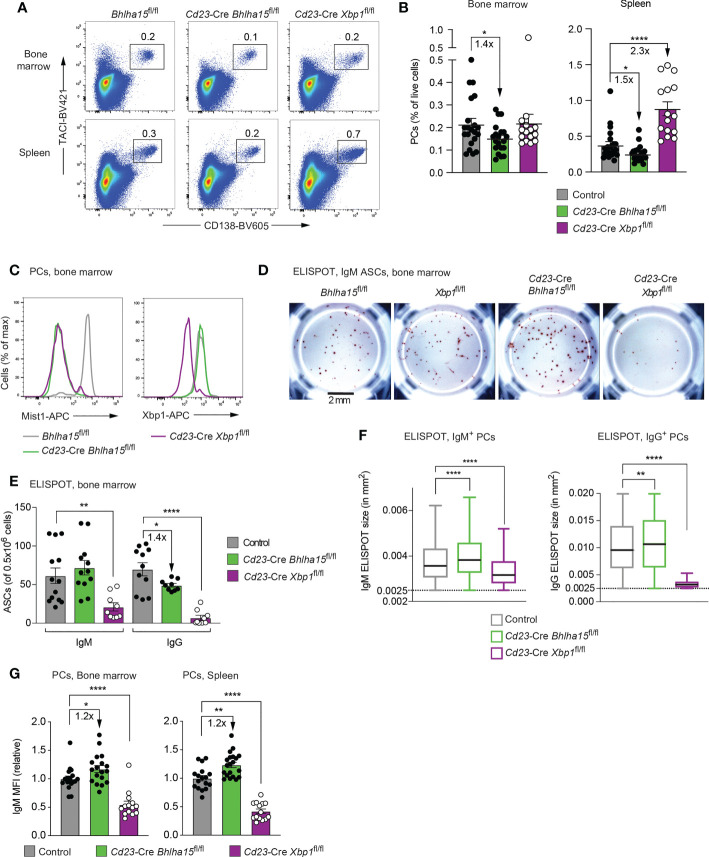
Reduced plasma cell numbers and increased antibody secretion in unimmunized *Cd23*-Cre *Bhlha15* ^fl/fl^ mice. **(A)** Flow-cytometric analysis of PCs (TACI^+^CD138^+^) in the spleen and bone marrow of 13-23-week-old mice of the indicated genotypes. The percentage of cells in the indicated gate is shown. One of 3 independent experiments with similar results is displayed. **(B)** The relative frequency of PCs in the bone marrow and spleen of 13-23-week-old mice of the indicated genotypes is shown for 3 independent experiments (*n* > 18 mice) per genotype. *Bhlha15* ^fl/fl^ and *Xbp1* ^fl/fl^ mice served as controls. **(C)** Expression of Mist1 and Xbp1 in PCs from the bone marrow of age-matched mice of the indicated genotypes, as determined by intracellular staining. One of two experiments is shown. **(D–F)** ELISPOT analysis of IgM and IgG antibody-secreting cells (ASC) from the bone marrow of age-matched mice of the indicated genotypes (see Methods). **(D)** Representative wells of an anti-IgM ELISPOT experiment. The scale bar indicates 2 mm. **(E)** Quantification of the number of IgM and IgG ASCs per 500,000 plated bone marrow cells. The combined data of two independent experiments are shown. **(F)** Size distribution of the antibody-containing dots, which were produced by the IgM and IgG ASCs shown in **(E)**. The dot sizes were automatically quantified by using the Fiji software (see Methods). Black lines indicate the median, and boxes represent the middle 50% of the data. Whiskers denote all values of the 1.5× interquartile range. Dots with a size of > 0.0025 mm^2^ are analyzed. The results of one of 2 independent experiments are shown. **(G)** Quantification of the mean fluorescence intensity (MFI) of intracellular IgM staining in plasma cells (TACI^+^CD138^+^) from the bone marrow (left) and spleen (right) of 13-23-week-old mice of the indicated genotypes. The MFI values of 3 independent experiments were normalized relative to those of the control mice (mean value set to 1). Statistical data are shown as mean values with SEM and were analyzed with the unpaired Student’s *t* test **(B–G)** or Mann-Whitney test **(F)**; *P < 0.05, **P < 0.01, ****P < 0.0001. Each dot **(B–G)** represents one mouse.

To study the function of Mist1 in PCs, we crossed *Bhlha15* ^fl/fl^ mice (35) with *Cd23*-Cre mice. In addition, we employed *Cd23*-Cre *Xbp1* ^fl/fl^ mice as reference mice with strongly impaired antibody secretion and used *Bhlha15* ^fl/fl^ and *Xbp1* ^fl/fl^ mice as controls. Consistent with the specific expression of *Bhlha15* in terminally differentiated PCs (**Supplementary Figure 2A**), B cell development was normal in *Cd23*-Cre *Bhlha15* ^fl/fl^ mice, as indicated by a similar frequency of mature B cells in the spleen of both experimental and control mice (**Supplementary Figure 2B**). However, flow cytometric analysis of PCs (TACI^+^CD138^+^) in unimmunized *Cd23*-Cre *Bhlha15* ^fl/fl^ mice revealed that the frequencies of PCs in the bone marrow and spleen were moderately (1.4-fold) reduced compared with those of control mice in three independent experiments (**Figures 2A, B**). In contrast, PCs in the spleen, but not in the bone marrow, were 2.3-fold increased in *Cd23*-Cre *Xbp1* ^fl/fl^ mice compared with control mice (**Figures 2A, B**). Intracellular staining combined with flow-cytometric analysis confirmed that Mist1 was lost in PCs from the bone marrow of both *Cd23*-Cre *Bhlha15* ^fl/fl^ and *Cd23*-Cre *Xbp1* ^fl/fl^ mice (**Figure 2C**). In the spleen, the Mist1 protein was also absent in *Cd23*-Cre *Bhlha15* ^fl/fl^ PCs, but was still lowly expressed in *Cd23*-Cre *Xbp1* ^fl/fl^ PCs (**Supplementary Figure 2C**). While the Xbp1 protein was lost in *Cd23*-Cre *Xbp1* ^fl/fl^ PCs in the bone marrow and spleen, it was similarly expressed in *Cd23*-Cre *Bhlha15* ^fl/fl^ and control PCs (**Figure 2C** and **Supplementary Figure 2C**). Hence, we conclude that Mist1 does not control *Xbp1* expression, while Xbp1 activates *Bhlha15* expression also in PCs. Notably, *Cd23*-Cre *Xbp1* ^fl/fl^ PCs were smaller in size than *Cd23*-Cre *Bhlha15* ^fl/fl^ and control PCs (**Supplementary Figure 2D**), which is likely caused by the low amount of ER in Xbp1-deficient PCs (12).

We next studied the antibody secretion of mutant PCs by performing ELISPOT analysis with bone marrow cells and found a small but significant decrease in IgG ASCs in *Cd23*-Cre *Bhlha15* ^fl/fl^ mice, while the number of IgM ASCs was comparable with that of control mice (**Figures 2D, E** and **Supplementary Figure 2E**). In contrast, IgG ASCs were almost lost, and IgM ASCs were significantly reduced in the bone marrow of *Cd23*-Cre *Xbp1* ^fl/fl^ mice (**Figure 2D, E**), consistent with the known role of Xbp1 in controlling antibody secretion (11, 12). Notably, the ELISPOT size of individual IgM or IgG ASCs was increased in *Cd23*-Cre *Bhlha15* ^fl/fl^ mice, suggesting that the antibody secretion was higher per mutant PC compared with a control PC (**Figure 2F**). To corroborate this result, we measured the IgM levels per plasma cell by intracellular flow-cytometric analysis and quantification of the mean fluorescence intensity (MFI), indicating that the expression of IgM was 1.2-fold increased in Bhlha15-deficient PCs compared with control PCs both in the spleen and bone marrow (**Figure 2G**). In this context, it is important to note that a 1.2-fold increase in IgM expression corresponds to a relatively strong transcriptional increase, as immunoglobulin transcripts account for up to 60% of all mRNAs in PCs (2, 3). Enzyme-linked immunosorbent assay (ELISA) revealed, however, normal titers of different antibody isoforms in the sera of *Cd23*-Cre *Bhlha15* ^fl/fl^ mice compared with control *Bhlha15* ^fl/fl^ mice (**Supplementary Figure 2F**). The normal antibody titers in *Cd23*-Cre *Bhlha15* ^fl/fl^ mice may be explained by the increased antibody secretion per PC (**Figures 2F, G**) that could compensate for the reduced PC numbers in these mice (**Figures 2A, B**). In contrast, *Cd23*-Cre *Xbp1* ^fl/fl^ mice had strongly reduced titers of all antibody isoforms compared with *Xbp1* ^fl/fl^ littermates (**Supplementary Figure 2F**). Together, these data indicate that the loss of *Bhlha15* leads to increased antibody secretion but lower numbers of plasma cells in the spleen and bone marrow.

### Mist1-dependent control of the plasma cell response to NP-KLH immunization

**Figure 3:**
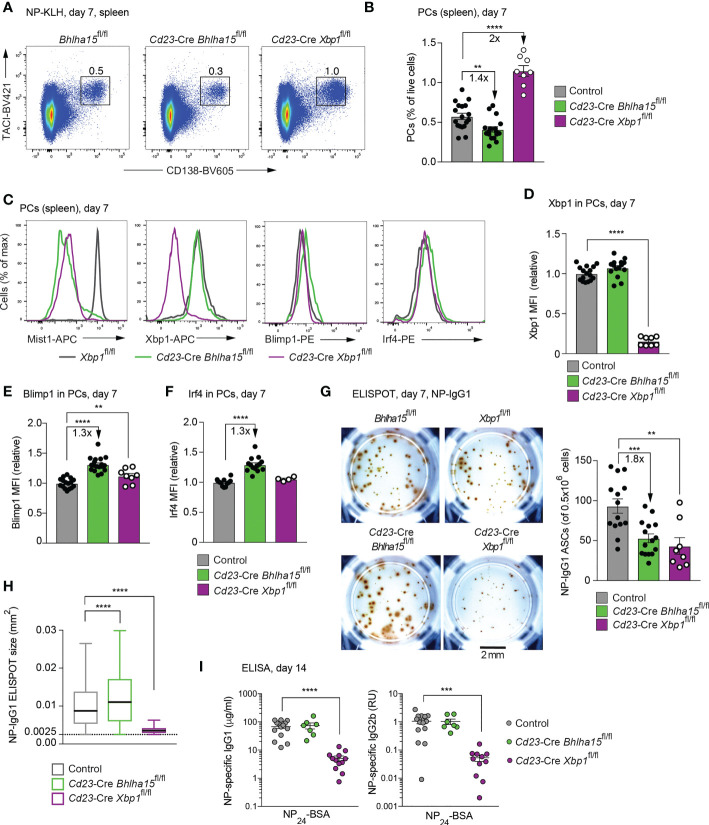
Reduced plasma cell numbers and increased antibody secretion in NP-KLH-immunized *Cd23*-Cre *Bhlha15* ^fl/fl^ mice. **(A)** Flow-cytometric analysis of PCs (TACI^+^CD138^+^) in the spleen of the indicated genotypes at day 7 after NP-KLH immunization (in alum). The percentage of cells in the indicated gate is shown. One of 2 (*Cd23*-Cre *Xbp1* ^fl/fl^) or 3 (*Cd23*-Cre *Bhlha15* ^fl/fl^ and control) independent experiments with similar results is displayed. **(B)** The relative frequency of PCs in the spleen of age-matched mice of the indicated genotypes at day 7 after NP-KLH immunization is shown for 2 or 3 independent experiments (see **A**). *Bhlha15* ^fl/fl^ and *Xbp1* ^fl/fl^ mice served as controls. **(C)** Expression of Mist1, Xbp1, Blimp1 and Irf4 in splenic PCs from mice of the indicated genotypes, as determined by intracellular staining at day 7 after NP-KLH immunization. **(D–F)** Quantification of the mean fluorescence intensity (MFI) of Xbp1 **(D)**, Blimp1 **(E)** and Irf4 **(F)** intracellular staining in PCs shown in **(C)**. The MFI values were normalized relative to those of the control mice (mean value set to 1). The results of 3 independent experiments are shown for the control and *Cd23*-Cre *Bhlha15* ^fl/fl^ genotypes and the results of 2 experiments are displayed for *Cd23*-Cre *Xbp1* ^fl/fl^ genotype. **(G)** ELISPOT analysis of NP-IgG1 ASCs from the spleen of the indicated genotypes at day 7 after NP-KLH immunization. Plates were coated with NP_24_-BSA and developed using anti-IgG1 antibodies. Left: Representative ELISPOT wells with the scale bar indicating 2 mm. Right: Quantification of the number of NP-IgG1 ASCs per 500,000 plated cells. The combined data of two experiments are shown. **(H)** Size distribution of the antibody-containing dots, which were produced by the NP-IgG1 ASCs shown in **(G)**. The dot sizes were automatically quantified by using the Fiji software (see Methods). Black lines indicate the median, and boxes represent the middle 50% of the data. Whiskers denote all values of the 1.5× interquartile range. Dots with a size of > 0.0025 mm^2^ are analyzed. The results of one of 2 experiments are shown. **(I)** Serum titers of NP-specific IgG1 or IgG2b antibodies at day 14 after NP-KLH immunization. Plates were coated with NP_24_-BSA and then developed with anti-IgG1 or anti-IgG2b. The NP-IgG1 concentration was determined relative to a NP-IgG1 standard (hybridoma line SSX2.1). RU; relative units. The data of 3 independent experiments are shown. Statistical data **(B, D-I)** are indicated as mean values with SEM and were analyzed with the unpaired Student’s *t* test **(B, D–G, I)** or Mann-Whitney test **(H)**; **P < 0.01; ***P < 0.001; ****, *P* < 0.0001. Each dot **(B, D–G, I)** represents one mouse.

To study the function of Mist1 in response to a defined antigen, we immunized mice with the T cell-dependent antigen NP-KLH (in alum) and analyzed the frequency of splenic PCs and immunoglobulin secretion at day 7 after immunization. The frequency of PCs (TACI^+^CD138^+^) was 1.4-fold reduced in the spleen of the *Cd23*-Cre *Bhlha15* ^fl/fl^ mice relative to control mice (**Figures 3A, B**). In contrast, the percentage of splenic PCs was 2-fold increased in *Cd23*-Cre *Xbp1* ^fl/fl^ mice compared with control mice (**Figures 3A, B**). As shown by intracellular staining at day 7 after immunization, the Mist1 protein was completely lost in *Cd23*-Cre *Bhlha15* ^fl/fl^ PCs and was only lowly expressed in *Cd23*-Cre *Xbp1* ^fl/fl^ PCs (**Figure 3C**). We next investigated the expression of the PC regulators Xbp1, Blimp1 and Irf4 by intracellular staining and MFI quantification. The Xbp1 protein was expressed at a similar level in *Cd23*-Cre *Bhlha15* ^fl/fl^ and control PCs (**Figures 3C, D**). In contrast, the expression of Blimp1 and Irf4 was 1.3-fold increased in *Cd23*-Cre *Bhlha15* ^fl/fl^ PCs relative to control PCs (**Figures 3C, E, F**).

ELISPOT analysis of splenocytes at day 7 after immunization revealed a decrease of NP-specific IgG1 ASCs in *Cd23*-Cre *Bhlha15* ^fl/fl^ and *Cd23*-Cre *Xbp1* ^fl/fl^ mice relative to control mice (**Figure 3G**). The ELISPOT size of NP-IgG1 ASCs was again increased in *Cd23*-Cre *Bhlha15* ^fl/fl^ mice and strongly decreased in *Cd23*-Cre *Xbp1* ^fl/fl^ compared with control mice (**Figure 3H**). The serum titers of NP-IgG1 and NP-IgG2b antibodies were also similar in *Cd23*-Cre *Bhlha15* ^fl/fl^ and control mice, possibly reflecting compensation between increased secretion and reduced frequency of the Mist1-deficient PCs, while the titers of both antibodies were strongly reduced in *Cd23*-Cre *Xbp1* ^fl/fl^ mice (**Figure 3I**), as expected (12, 13).

In summary, our analysis of PCs *in vivo* in unimmunized or NP-KLH-immunized mice demonstrated that the loss of Mist1 resulted in decreased PC numbers but increased antibody secretion per PC.

### Normal *in vitro* differentiation and antibody secretion of plasmablasts lacking *Bhlha15*

**Figure 4:**
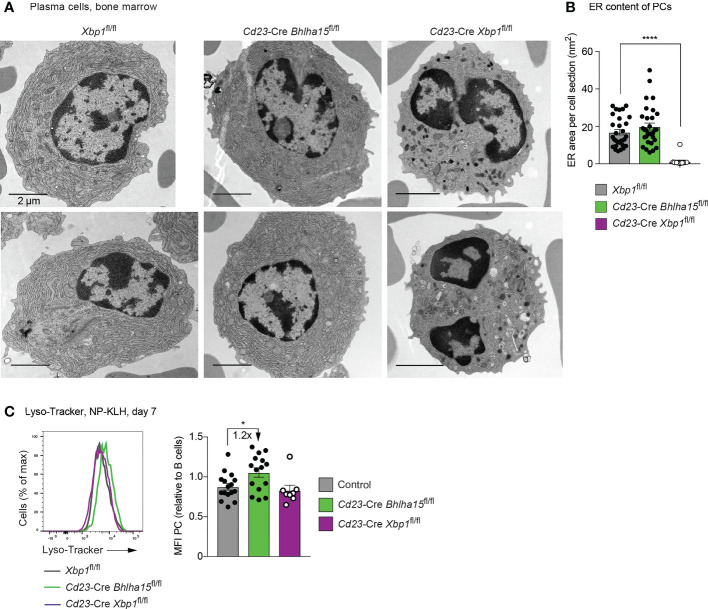
Normal morphological structure of *Cd23*-Cre *Bhlha15* ^fl/fl^ plasma cells. **(A)** Electron microscopic analysis of bone marrow PCs of the indicated genotypes, which were sorted as B220^int^CD28^+^CD138^+^Lin^–^ cells, fixed and processed as described in Methods. The scale bars indicate 2 µm. The cells surrounding the PCs are erythrocytes. **(B)** Quantification of the content of endoplasmic reticulum (ER) in PCs of the different genotypes. The ER content was manually determined by using the Fiji software. Each dot represents one PC. In total, 33 *Xbp1* ^fl/fl^ PCs, 14 *Cd23*-Cre *Xbp1* ^fl/fl^ PCs, and 31 *Cd23*-Cre *Bhlha15* ^fl/fl^ PCs were analyzed. **(C)** Staining of splenic PCs (TACI^+^CD138^+^) of the indicated genotypes with Lyso-Tracker at day 7 after NP-KLH immunization (left). The MFI values of the Lyso-Tracker staining (right) are shown for all PCs analyzed in 3 independent experiments. Each dot represents one mouse. Statistical data **(B, C)** are shown as mean values with SEM and were analyzed with the unpaired Student’s *t* test; *P < 0.05; ****P < 0.0001.

As the *in vivo* phenotype of the *Bhlha15* mutant PCs is at odds with the reported absence of phenotypic differences between LPS-stimulated PBs derived from wild-type and *Bhlha15* ^–/–^ B cells (33), we reinvestigated a possible role of Mist1 in *in vitro* differentiated PBs. To this end, we analyzed PBs after 4 days of treatment with LPS and IL-4 (**Supplementary Figures 3A–C**) or after 8 days of stimulation in the iGB system (**Supplementary Figures 3D, E**). LPS plus IL-4 stimulation of B cells from *Cd23*-Cre *Bhlha15* ^fl/fl^ and control mice resulted in the generation of similar numbers of PBs and IgM ASCs, which were furthermore characterized by a similar ELISPOT size (**Supplementary Figures 3A–C**). Likewise, *Cd23*-Cre *Bhlha15* ^fl/fl^ and control B cells gave rise to similar numbers of IgE ASCs with a similar ELISPOT size after 8 days in the iGB system (**Supplementary Figures 3D, E**). While *Cd23*-Cre *Xbp1* ^fl/fl^ PBs were generated in similar numbers as control PBs upon stimulation with LPS and IL-4 (**Figure 4A**), they exhibited a severe defect in antibody secretion in both differentiation systems, as shown by the reduced numbers of ASCs and their smaller ELISPOT size, consistent with published data (12). We conclude therefore that *in vitro* PB differentiation did not recapitulate the *in vivo* phenotype of *Cd23*-Cre *Bhlha15* ^fl/fl^ PCs, as the respective *in vitro* differentiated PBs were generated in normal numbers and exhibited normal antibody secretion.

### Normal cellular morphology of Mist1-deficient plasma cells *in vivo*

We next analyzed the cellular architecture of *in vivo* PCs by electron microscopy (EM). For this, we sorted PCs as Lin^–^B220^int^CD138^hi^CD28^+^ cells from the bone marrow of unimmunized mice by flow cytometry prior to EM analysis. The cytoplasm of *Cd23*-Cre *Xbp1* ^fl/fl^ PCs contained little ER, which was thin and disorganized (**Figures 4A, B** and **Supplementary Figure 4A**). By measuring the ER mass per section, we confirmed a significant reduction of the ER in the *Cd23*-Cre *Xbp1* ^fl/fl^ PCs relative to *Xbp1* ^fl/fl^ PCs (**Figure 4B**), which is consistent with the observed small size of Xbp1-deficient PCs (**Supplementary Figure 2D**). A previous EM analysis of *Cd19*-Cre *Xbp1* ^fl/fl^ PCs demonstrated that, in addition to the low ER content, the Xbp1-deficient plasma cells also contained large vesicular structures (12) that we did, however, not detect in our analysis. The *Cd23*-Cre *Bhlha15* ^fl/fl^ and control PCs had the same large size and a similarly extended network of well-stacked ER, whose cytosolic surfaces were densely occupied with ribosomes (**Figure 4A** and **Supplementary Figure 4A**). As shown by quantification of the ER mass, the *Cd23*-Cre *Bhlha15* ^fl/fl^ and control PCs had a similar content of ER (**Figure 4B**). Moreover, at day 7 after NP-KLH immunization, splenic PCs of *Cd23*-Cre *Bhlha15* ^fl/fl^ and control mice were equally stained with a Golgi-Tracker or ER-Tracker fluorescent dye (**Supplementary Figures 4B, C**). Based on these data, we conclude that the *Cd23*-Cre *Bhlha15* ^fl/fl^ and control PCs have a similar ultrastructure. Upon staining with the Lyso-Tracker fluorescent dye, the *Cd23*-Cre *Bhlha15* ^fl/fl^ PCs exhibited a 1.2-fold increase in staining intensity compared to control PCs (**Figure 4C**), which indicates an increase of acidic compartments in the absence of Mist1. The *Cd23*-Cre *Xbp1* ^fl/fl^ PCs in contrast displayed significantly reduced Golgi-Tracker and ER-Tracker staining (**Supplementary Figures 4B, C**), while the Lyso-Tracker staining was comparable to that of control cells (**Figure 4C**).

As changes in ER and Golgi structures can lead to altered glycosylation patterns of antibodies (36), we analyzed the glycoprofiles by assessing the relative abundance of IgG3-specific glycopeptide glycoforms purified from mouse serum by liquid chromatography electrospray ionization mass spectrometry (LC-ESI-MS). IgG3 peptides from *Cd23*-Cre *Xbp1* ^fl/fl^ mice exhibited altered glycoprofiles defined by reduced fucosylation (GnGnF) and increased sialylation (NgAF, NgNgF), when compared to IgG3 peptides from *Cd23*-Cre *Bhlha15* ^fl/fl^ and control mice (**Supplementary Figure 2G**). Additionally, the sera of *Cd23*-Cre *Xbp1* ^fl/fl^ mice contained non-fucosylated GnGn and AGn glycoforms, which were absent in the sera of *Cd23*-Cre *Bhlha15* ^fl/fl^ and control mice (**Supplementary Figure 2G**). Hence, the glycosylation of antibodies is altered in *Cd23*-Cre *Xbp1* ^fl/fl^ PCs consistent with the observed defect in ER structure. The similar glycosylation pattern of IgG3 in *Cd23*-Cre *Bhlha15* ^fl/fl^ and control PCs further demonstrates that the ER and Golgi structures are functional in the absence of Mist1.

### Mist1-binding regions overlap with E2A peaks in plasmablasts

**Figure 5:**
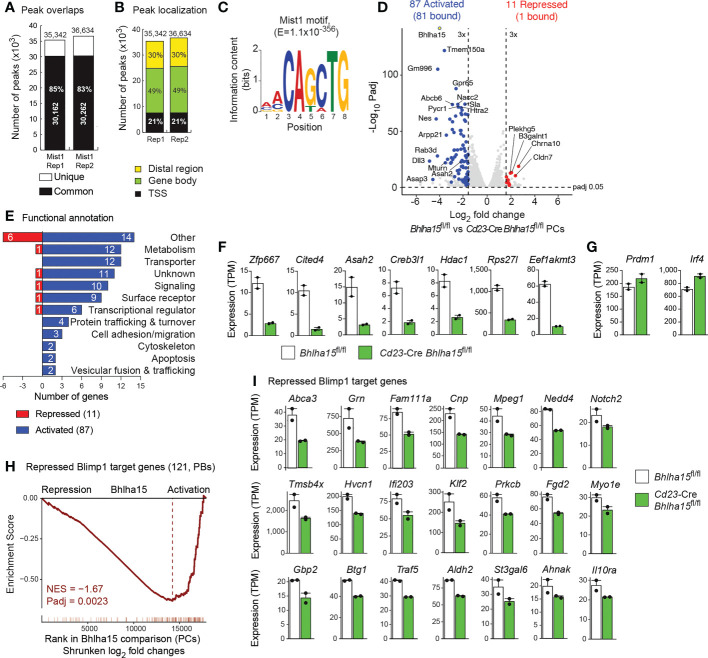
Mist1-dependent gene expression program in plasma cells. **(A–C)** Genome-wide Mist1 binding in PBs that were *in vitro* differentiated for 4 days by LPS stimulation of CD43^–^ B cells from the spleen of *Bhlha15* ^fl/fl^ *Rosa26* ^BirA/BirA^ mice. CD138^+^ PBs, which were purified by immunomagnetic enrichment with CD138-MicroBeads, were used for Bio-ChIP-seq analysis (22), and Mist1 peaks were identified by MACS peak calling with a stringent *P* value of < 10^–10^. **(A)** Overlap and number of Mist1 peaks identified in two Bio-ChIP-seq replica (Rep) experiments. **(B)** Presence of Mist1 peaks in distal regions, gene bodies and promoter (TSS) regions. Mist1 peaks were assigned to genes, as described (22). **(C)** Consensus Mist1-binding motif identified with an E-value of 1 x 10^-356^ by the *de novo* motif-discovery program MEME-Chip. **(D)** Volcano plot of gene expression differences between *Cd23-*Cre *Bhlha15* ^fl/fl^ and control *Bhlha15* ^fl/fl^ PCs that were sorted as TACI^+^CD138^+^ cells from the spleen at day 7 after NP-KLH immunization. Two independent RNA-seq experiments were performed with PCs of each genotype. Each dot corresponds to one gene, whose expression is plotted as log_2_-fold change against the -log_10_ adjusted *P* value. Genes with an expression difference of > 3-fold, an adjusted *P* value of < 0.05 and an expression value of > 5 TPM (at least in one sample) are colored in blue or red corresponding to activation or repression by Mist1, respectively. **(E)** Functional classification and quantification (number) of proteins encoded by Mist1-activated and Mist1-repressed genes. **(F)** Expression of selected activated Mist1 target genes in *Cd23-*Cre *Bhlha15* ^fl/fl^ and *Bhlha15* ^fl/fl^ PCs. **(G)** Expression of *Prdm1* and *Irf4* in *Cd23-*Cre *Bhlha15* ^fl/fl^ and *Bhlha15* ^fl/fl^ PCs. **(H)** GSEA analysis of 121 repressed Blimp1 target genes (3), as compared to their ranked shrunken log_2_-fold gene expression changes in *Cd23-*Cre *Bhlha15* ^fl/fl^ PCs versus *Bhlha15* ^fl/fl^ PCs. NES, normalized enrichment score. **(I)** Expression of repressed Blimp1 target genes in *Bhlha15* ^fl/fl^ and *Cd23-*Cre *Bhlha15* ^fl/fl^ PCs, as determined by RNA-seq. All depicted genes are significantly deregulated with an adjusted *P* value of < 0.05.

We next performed RNA- and ChIP-seq experiments to investigate the role of Mist1 in PCs. For ChIP-seq analysis, we took advantage of the fact that the floxed *Bhlha15* allele contains an N-terminal insertion of a biotin acceptor sequence (35). Splenic B cells from *Bhlha15* ^fl/fl^ *Rosa26* ^BirA/BirA^ mice were stimulated with LPS for 4 days and then enriched for CD138^+^ PBs by immunomagnetic sorting (**Supplementary Figure 5A**), followed by Bio-ChIP-seq analysis (22). Peak calling of the data of two Bio-ChIP-seq experiments with a *P* value of < 10^–10^ identified 35,342 and 36,634 Mist1 peaks, resulting in an overlap of 30,212 common peaks that were used for subsequent analysis (**Figure 5A**). A large fraction of the Mist1 peaks was located in gene bodies (49%; **Figure 5B**), indicating that Mist1 binds less frequently to TSS regions (21%) relative to Xbp1 (38%) (**Figure 1D**). *De novo* motif discovery analysis of the Mist1 peak sequences identified a consensus Mist1-binding motif (**Figure 5C**) that resembles the consensus binding motif of the bHLH transcription factor E2A (**Supplementary Figure 5B**) (27). We next overlapped the Mist1 peaks with the 11,872 E2A peaks that we previously identified in LPS-differentiated PBs (27). Notably, 94% of all E2A peaks overlapped with Mist1 peaks (**Supplementary Figure 5C**), suggesting that the Mist1 homodimers may bind to the same recognition sequences as E2A homodimers or E2A-E2-2 heterodimers in PBs. Alternatively, it is conceivable that Mist1 forms heterodimers with E2A in PBs, as previously shown in a myoblast cell line (37). Streptavidin pulldown of biotinylated Mist1 from nuclear extracts of LPS-differentiated *Bhlha15* ^fl/fl^ *Rosa26* ^BirA/BirA^ PBs revealed that E2A was co-precipitated with Mist1, indicating that both bHLH proteins can form heterodimers also in PBs (**Supplementary Figure 5D**). Consistent with this finding, co-binding of Mist1 and E2A was observed in PBs at many previously identified elements (27) of the *Prdm1* (Blimp1) locus (**Supplementary Figure 5G**). Together, these data suggest that Mist1 and E2A can bind their genomic recognition sequences as heterodimers in PBs.

### Mist1 and Xbp1 regulate largely distinct gene expression programs in plasma cells

We next performed RNA-seq analysis with *ex vivo* sorted TACI^+^CD138^+^ PCs from the spleen of *Bhlha15* ^fl/fl^ and *Cd23*-Cre *Bhlha15* ^fl/fl^ mice 7 days after NP-KLH immunization. Comparison of the two RNA-seq datasets identified 87 Mist1-activated and 11 Mist1-repressed genes, which were selected for an expression difference of > 3-fold, an adjusted *P* value of < 0.05 and an expression value of > 5 TPM in *Bhlha15* ^fl/fl^ PCs (activated genes) or *Cd23*-Cre *Bhlha15* ^fl/fl^ PCs (repressed genes), respectively (**Figure 5D** and **Supplementary Figure 5E**, **Supplementary Table 2**). Interestingly, 81 of the 87 activated genes were bound by Mist1 in contrast to only 1 of the 11 repressed genes, indicating that Mist1 mainly functions a transcriptional activator in PCs. The most prominent functional classes of Mist1-activated genes code for 12 metabolic proteins, 12 transporters and 10 signaling molecules, while genes implicated in UPR and ER function were not regulated by Mist1 (**Figure 5E**). Seven activated Mist1 target genes code for transcriptional (Zfp667, Cited4, Creb3l1, Hdac1) and translational (Rps27l, Eed1akmt3) regulators as well as for the autophagy-controlling Asah2 protein (**Figure 5F**). Similar to the observed increase of Blimp1 and Irf4 protein expression (**Figures 3E, F**), the *Prdm1* and *Irf4* mRNA expression was also moderately upregulated in Mist1-deficient PCs (**Figure 5G**), consistent with Mist1 binding at both genes (**Supplementary Figures 5F, G**). Moreover, only 8 genes were deregulated more than 3-fold in both Mist1-deficient PCs and Xbp1-deficient PBs (**Supplementary Figure 5H**), indicating that both factors largely regulate distinct gene expression programs in PBs.

As Blimp1 (*Prdm1*) expression was increased in Mist1-deficient PCs (**Figures 3E, 5G**), we next used the previously identified 121 repressed Blimp1 target genes (3) for gene set enrichment analysis (GSEA) of the RNA-seq data of control and Mist1-deficient PCs. Interestingly, there was a strong correlation between repressed Blimp1 target genes and Mist1-activated genes (**Figure 5H**). Importantly however, there was no overlap between the published repressed Blimp1 target genes (> 3-fold) (3) and the Mist1-activated genes (> 3-fold; **Figure 5D** and **Supplementary Table 2**). In contrast, the expression of most Blimp1-repressed target genes (3) was modestly (≤ 2-fold) reduced in *Cd23*-Cre *Bhlha15* ^fl/fl^ PCs compared with control *Bhlha15* ^fl/fl^ PCs (**Figure 5I**). This repression is likely caused by the increased expression of Blimp1 in *Cd23*-Cre *Bhlha15* ^fl/fl^ PCs and may thus not reflect direct activation by Mist1. In summary, we conclude that Mist1 regulates a largely different gene expression program than Xbp1 and further represses Blimp1 target genes by activating *Prdm1* expression in PCs.

### Reduced Blimp1 expression rescues the cell number and antibody secretion of Mist1-deficient plasma cells

**Figure 6:**
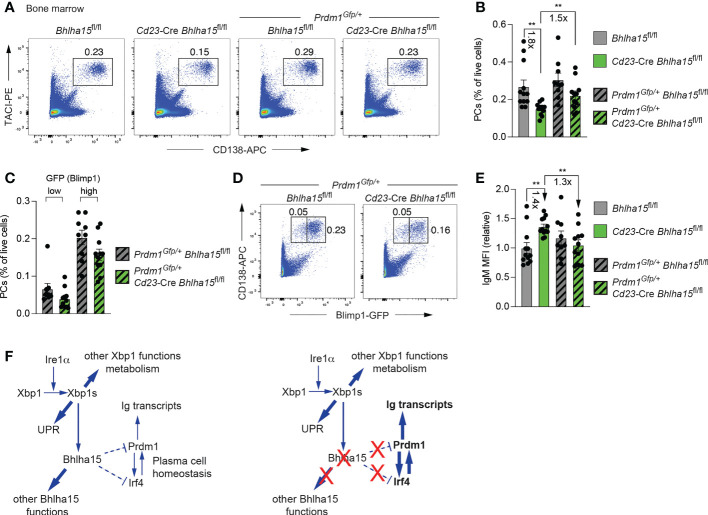
Reduced Blimp1 expression rescues the cell number and antibody secretion of Mist1-deficient plasma cells. **(A)** Flow-cytometric analysis of PCs (TACI^+^CD138^+^) in the bone marrow of unimmunized 10-13-week-old mice of the indicated genotypes. The percentage of cells in the gate is indicated. One of two independent experiments is shown. **(B)** Frequency of bone marrow PCs in unimmunized mice of the indicated genotypes. The frequencies were calculated based on the data of two independent experiments performed with *Bhlha15* ^fl/fl^ (*n* = 12), *Cd23*-Cre *Bhlha15* ^fl/fl^ (*n* = 12), *Prdm1* ^Gfp/+^ *Bhlha15* ^fl/fl^ (*n* = 9) and *Prdm1* ^Gfp/+^ *Cd23*-Cre *Bhlha15* ^fl/fl^ (*n* = 11) mice. **(C, D)** Flow cytometric analysis of GFP (Blimp1) expression in PCs (GFP^+^CD138^+^) from the bone marrow of *Prdm1* ^Gfp/+^ *Cd23*-Cre *Bhlha15* ^fl/fl^ and control *Prdm1* ^Gfp/+^ *Bhlha15* ^fl/fl^ mice **(D)**. Frequency of GFP^hi^ and GFP^lo^ PCs in the bone marrow of *Prdm1* ^Gfp/+^ *Cd23*-Cre *Bhlha15* ^fl/fl^ and *Prdm1* ^Gfp/+^ *Bhlha15* ^fl/fl^ mice **(C)**. **(E)** Quantification of the mean fluorescence intensity (MFI) of intracellular IgM staining in bone marrow PCs (TACI^+^CD138^+^) of unimmunized mice with the genotypes and mouse numbers described in **(B)**. The MFI data are shown relative to the mean value of the *Bhlha15* ^fl/fl^ PCs (set to 1). **(F)** Model explaining how the loss of *Bhlha15* (Mist1) influences the regulatory networks controlling PC homeostasis and antibody secretion. Statistical data are shown as mean values with SEM and were analyzed with the unpaired Student’s *t* test (**B**, **C**, **E**); **P < 0.01. Each dot **(B, C, E)** represents one mouse.

Blimp1 is known to contribute to antibody secretion by strongly activating the transcription of the *Igh* and *Igk* genes and by regulating the posttranscriptional expression switch from the membrane-bound form to the secreted form of the Ig heavy chain (3). Hence, it is conceivable that the increased Blimp1 expression in *Cd23*-Cre *Bhlha15* ^fl/fl^ PCs may be responsible for the observed increase in antibody secretion in addition to the moderate repression of its target genes. To test this hypothesis, we took advantage of the *Prdm1* ^Gfp^ null allele (38) to reduce the expression of Blimp1 by a factor of two in bone marrow PCs of unimmunized *Prdm1* ^Gfp/+^ *Cd23*-Cre *Bhlha15* ^fl/fl^ mice. While the frequency of PCs was 1.8-fold reduced in *Cd23*-Cre *Bhlha15* ^fl/fl^ mice compared to control *Bhlha15* ^fl/fl^ mice, it was 1.5-fold increased in *Prdm1* ^Gfp/+^ *Cd23*-Cre *Bhlha15* ^fl/fl^ mice relative to *Cd23*-Cre *Bhlha15* ^fl/fl^ mice and thus reached a similar level observed in control *Bhlha15* ^fl/fl^ and *Prdm1* ^Gfp/+^ *Bhlha15* ^fl/fl^ mice (**Figures 6A, B**). Moreover, the frequencies of both the less mature GFP(Blimp1)^lo^ and more mature GFP(Blimp1)^hi^ PCs (38) were equally but only moderately reduced in *Prdm1* ^Gfp/+^ *Cd23*-Cre *Bhlha15* ^fl/fl^ compared to control *Prdm1* ^Gfp/+^ *Bhlha15* ^fl/fl^ mice (**Figures 6C, D**). Importantly, the expression of IgM was 1.4-fold increased only in PCs of *Cd23*-Cre *Bhlha15* ^fl/fl^ PCs but not in PCs of *Prdm1* ^Gfp/+^ *Cd23*-Cre *Bhlha15* ^fl/fl^ mice or the control *Prdm1* ^Gfp/+^ *Bhlha15* ^fl/fl^ and *Bhlha15* ^fl/fl^ mice, as shown by intracellular staining (**Figure 6E**). Hence, a 2-fold reduction of Blimp1 expression restored both PC numbers and IgM expression in *Prdm1* ^Gfp/+^ *Cd23*-Cre *Bhlha15* ^fl/fl^ mice. These data therefore indicate that Mist1 largely mediates its effects on plasma cells by restraining Blimp1 expression.

## Discussion

An essential function of the transcription factor Xbp1 is to activate the UPR gene expression program upon induced ER stress in most cell types (39) and to promote antibody secretion at a high rate in PBs and PCs of the B cell lineage (7, 11, 12). By analyzing the Xbp1-dependent gene expression program in PBs, we identified the *Bhlha15* (Mist1) gene as the most strongly activated direct target gene of Xbp1. Mist1 was previously shown to play an important role in other secretory cell types by inducing and maintaining their secretory cell architecture (15–18). By systematically investigating the phenotype of Mist1-deficient PCs *in vivo* in unimmunized and NP-KLH-immunized mice, we realized that antibody secretion was moderately increased in Mist1-deficient PCs, in marked contrast to the strong reduction of antibody secretion in Xbp1-deficient PCs (12, 13). Although Mist1 is also not expressed in the absence of Xbp1, the loss of immunoglobulin secretion in Xbp1-deficient PCs interferes with the manifestation of the Mist1-deficient phenotype, which appears to be largely caused by increased immunoglobulin secretion. Molecular analyses revealed that Mist1 and Xbp1 regulate largely different sets of target genes, as Xbp1 regulates UPR genes involved in ER expansion and antibody secretion, while Mist1 restrains the expression of Blimp1 and Irf4 in PCs.

A previous study investigating the role of Mist1 in LPS-stimulated *Bhlha15* ^–/–^ PBs (33) did not report any differences in the formation and function of mutant and wild-type PBs, which is consistent with our finding that *Cd23*-Cre *Bhlha15* ^fl/fl^ and control B cells differentiated *in vitro* to PBs at equal frequency in response to LPS stimulation or treatment with IL-21 in the iGB system. A second study also did not find significant differences in the generation and function of splenic PCs *in vivo* by analyzing few immunized *Bhlha15* ^–/–^ and wild-type mice (34). In our comprehensive study, we performed multiple experiments to analyze *in vivo* PCs in the spleen and bone marrow of *Cd23*-Cre *Bhlha15* ^fl/fl^ and control mice under steady-state condition and upon immunization, which allowed us to detect a significant decrease in PCs and a significant increase in antibody secretion in the *Bhlha15* mutant mice.

In the absence of Mist1, PCs were not only reduced in number but also secreted more antibodies per cell, based on their increased immunoglobulin protein expression and larger ELISPOT size. Consistent with efficient antibody secretion, Mist1-deficient PCs had a similar cell size and content of well-stacked rough ER as control PCs. While a previous study reported subtle difference in the ER structure of two analyzed Mist1-deficient PCs in the small intestine (33), we provide now statistically relevant data for our observation that Mist1-deficient PCs in the bone marrow have a normal ER ultrastructure.

The enhanced antibody production and secretion in Mist1-deficient PCs likely increases the ER stress, which may impair cell survival, thus explaining the reduced PC numbers in *Cd23*-Cre *Bhlha15* ^fl/fl^ mice. In this context, it is important to mention that autophagy is known to restrain antibody secretion in PCs, thereby promoting homeostasis and survival of PCs (40). Autophagy-deficient PBs show increased antibody secretion, Blimp1 expression and apoptosis, similar to the Mist1-deficient PCs. Interestingly, Lyso-Tracker staining of Mist1-deficient PCs revealed an increase in acidic compartments consisting of lysosomes or autophagosomes. The Mist1-activated gene *Asah2* (nCDase) has been implicated in the control of autophagy, as its inactivation in mouse embryonic fibroblasts results in increased autophagy (41). The downregulation of this gene in Mist1-deficient PCs (**Figure 5F**) may explain the observed increase in acidic compartments, which likely indicates enhanced autophagy. This observation therefore suggests that enhanced autophagy is not able to correct the Blimp1-induced increase of antibody secretion in Mist1-deficient PCs.

The elevated expression of the PC-specific transcription factor Blimp1 is likely responsible for the increased antibody secretion by Mist1-deficient PCs, as Blimp1 is known to strongly activate the transcription of the *Igh* and *Igk* genes *via* their 3’ enhancer and to regulate the posttranscriptional expression switch from the membrane-bound form to the secreted form of the Ig heavy chain (3) (**Figure 6F**). In addition to Blimp1, the expression of Irf4, another essential PC regulator, is also increased in Mist1-deficient PCs. Irf4 and Blimp1 appear to cross-regulate each other, as Irf4 activates *Prdm1* (Blimp1) expression at the onset of PB differentiation (5, 6), while Blimp1 further induces *Irf4* expression in PBs (3) (**Figure 6F**). Both *Irf4* and *Prdm1* are bound by Mist1 and thus qualify as potentially repressed Mist1 target genes, although DNA-binding data alone can only suggest, but not prove, a direct role of Mist1 in the repression of *Prdm1*, *Irf4* or both genes (**Figure 6F**). Importantly, a 2-fold reduction of Blimp1 expression was sufficient to restore both PC numbers and antibody expression in *Prdm1* ^Gfp/+^ *Cd23*-Cre *Bhlha15* ^fl/fl^ mice. These genetic data therefore demonstrate that Mist1 largely mediates its effects on plasma cells by restraining Blimp1 expression (**Figure 6F**).

Mist1 lacks a transactivation domain (14), and its homodimer is therefore considered to act as a transcriptional repressor (37). However, Mist1 predominantly functions as a transcriptional activator in PCs, as shown by our transcriptomic analysis. Here, we demonstrate that Mist1 can heterodimerize with the E-protein E2A in PBs, as it was previously shown in a myoblast cell line (37). Moreover, the majority of all E2A-binding sites in PBs (27) were also bound by Mist1, suggesting that Mist1 may bind DNA as an Mist1-E2A heterodimer in PBs. As E2A contains two strong transactivation domains (42, 43), it is likely that Mist1 can activate gene expression in PCs primarily by recruiting E2A as a Mist1-E2A heterodimer to the activated Mist1 target genes.

In summary, our study has identified Mist1 as a critical regulator that restrains Blimp1 expression and thus reduces antibody secretion to promote PC viability similar to the role of autophagy in PCs (40).

## Data availability statement

The RNA-seq and ChIP-seq data, which were generated for this study (**Supplementary Table 3**), are available at the Gene Expression Omnibus (GEO) repository under the accession numbers GSE190591.

## Ethics statement

The animal study was reviewed and approved by the Magistratsabteilung 58, Amt der Wiener Landesregierung, City of Vienna.

## Author contributions

MW performed most experiments, TP performed the flow-cytometric analyses of unimmunized PCs, the intracellular staining of IgM expression and the Blimp1 rescue experiments, PB performed the Xbp1 ChIP-seq experiment, AH and JS performed the glycosylation analysis, DK-P generated the *Xbp1* ^Bio/+^ mouse, SK generated the *Bhlh15* ^fl/fl^ mouse, MJ and MF performed the bioinformatic analysis of the RNA-seq and ChIP-seq data, respectively, MB and MW planned the project and wrote the manuscript. All authors contributed to the article and approved the submitted version.
