# Supplementary material for: The Xbp1-regulated transcription factor Mist1 restricts antibody secretion by restraining Blimp1 expression in plasma cells
Source: Front Immunol. 2022 Dec 21;13:859598. doi: 10.3389/fimmu.2022.859598 (PMC9811352; doi:10.3389/fimmu.2022.859598)
Supplement: Supplementary file 4 [file DataSheet_1.pdf]

Supplementary Figure Legends

Wöhner et al.

SUPPLEMENTARY FIGURE 1 | Analysis of XBP1-dependent gene regulation.

(A) Flow-cytometric sorting strategy for isolating PBs used for RNA-seq analysis. Cells were stimulated in the iGB system for 4 days on 40LB feeder cells with IL-4 followed by 4 days of IL-21 stimulation and subsequent sorting of $CD19^{+}CD138^{+}CD23^{-}$ PBs. **(B)** Principal component analysis based on 2 RNA-seq experiments performed with PBs of each indicated genotype. **(C)** Gene set enrichment analyses of 408 Xbp1-activated genes (left) and 108 UPR genes (right), both defined in PCs (*1*), as compared to the their ranked shrunken log_2-fold gene expression changes in *Cd23*-Cre *Xbp1*$^{fl/fl}$ versus *Xbp1*$^{fl/fl}$ PBs. NES, normalized enrichment score; Padj, adjusted *P* value. **(D)** Expression of genes coding for important PC transcription factors, shown as mean transcript per million (TPM) values of two RNA-seq experiments of *Cd23*-Cre *Xbp1*$^{fl/fl}$ or *Xbp1*$^{fl/fl}$ PBs, respectively. **(E)** Schematic diagram of the Xbp1s-Bio protein encoded by the *Xbp1s*^{Bio} allele. The C-terminal tag sequence added at the last codon of Xbp1s contained a TEV protease cleavage site, epitopes for anti-V5 and anti-Flag antibodies, a PreScission (PreS) protease cleavage site and a biotin acceptor sequence (Bio) for biotinylation by the *E.coli* ligase BirA. **(F)** Normal B cell development in the bone marrow (left) and spleen (right) of *Xbp1*$^{Bio/Bio}$ *Rosa26*$^{BirA/BirA}$ mice compared with *Rosa26*$^{BirA/BirA}$ mice, as shown by flow-cytometric analysis. Imm, immature; mat, mature; FO, follicular; MZ, marginal zone. Each dot represents one mouse. One experiment is shown. **(G)** Mist1 protein expression in PCs from the bone marrow of *Xbp1*$^{Bio/Bio}$ *Rosa26*$^{BirA/BirA}$ and *Rosa26*$^{BirA/BirA}$ mice, as determined by intracellular staining with an anti-Mist1 antibody. The mean fluorescence intensity (MFI) of the intracellular Mist1 staining is shown for one experiment. **(H)** ELISPOT analysis of bone marrow cells from unimmunized *Xbp1*$^{Bio/Bio}$ *Rosa26*$^{BirA/BirA}$ and *Rosa26*$^{BirA/BirA}$ mice. Plates were coated and developed with antibodies detecting the indicated immunoglobulin isoforms. ASC, antibody-secreting cells. **(I)** Published consensus Xbp1-binding motif (*2*). Statistical data (**F**-**H**) are shown a mean value with SEM and were analyzed with the unpaired Student's *t* test.

SUPPLEMENTARY FIGURE 2 | Analysis of plasma cells in *Cd23*-Cre *Bhlha15*$^{fl/fl}$ and *Cd23*-Cre *Xbp1*$^{fl/fl}$ mice. **(A)** Expression of *Bhlha15* mRNA, shown as transcripts per million (TPM) value determined by RNA-seq analysis of the indicated B cell populations. Mature B cells of *Prdm1*$^{Gfp/+}$ mice were cultured in the iGB system for 6 days with IL-4 followed by 3 days with IL-21. Activated B cells (Act B) were sorted as $CD19^{+}$Blimp1-$GFP^{-}CD138^{-}$ cells, pre-plasmablasts (pre-PB) as $CD19^{+}$Blimp1-$GFP^{+}CD138^{-}$ cells and plasmablasts (PB) as $CD19^{+}$Blimp1-$GFP^{+}CD138^{+}$ cells. Germinal center (GC) B cells were sorted as $B220^{+}CD19^{+}GL7^{+}Fas^{+}$ cells at day 14 after immunization with sheep red blood cell. The RNA-seq data of mature B cells and plasma cells were previously published (*3, 4*). **(B)** Frequency of mature B cells in the spleen of the indicated genotypes, as shown by flow-cytometric analysis. *Bhlha15*$^{fl/fl}$ and *Xbp1*$^{fl/fl}$ mice served as controls. **(C)** Intracellular staining of Mist1 and Xbp1 expression in splenic PCs of the indicated genotypes. One of 3 experiments is shown. **(D)** Flow-cytometric analysis of the cell size (FCS-A) of PCs from the bone marrow of unimmunized mice of the indicated genotypes. **(E)** ELISPOT

analysis of IgG antibody-secreting cells (ASC) from the bone marrow of unimmunized age-matched mice of the indicated genotypes. Representative wells of an anti-IgG ELISPOT experiment are shown together with a scale bar of 2 mm. **(F)** Serum titers of total antibodies of the IgM, IgG1, IgG2b, IgG3, IgA and IgE isotypes in unimmunized 10-11-week-old mice of the indicated genotypes, as determined by ELISA (see Methods). The sera of two experiments were analyzed. **(G)** Schematic representation of a glycan tree attached to Asn297 of the IgG3 heavy chain (right). The glycan tree contains monomers of N-acetylglucosamine (Gn), fucose (F), mannose (M), galactose (A) and sialic acid (Ng). The relative abundance of the different IgG3 isoforms is shown (left). One experiment was performed. Statistical data (**B**, **F**, **G**) are shown as mean values with SEM and were analyzed with the unpaired Student's *t* test; *$P < 0.05$; **$P < 0.01$; ***$P < 0.001$. Each dot (**B**, **F**, **G**) represents one mouse.

SUPPLEMENTARY FIGURE 3 | Mist1 is dispensable for antibody secretion by *in vitro* differentiated plasmablasts. **(A-C)** Analysis of PBs that were generated from splenic CD43^{-} B cells of the indicated genotypes by *in vitro* stimulation with LPS and IL-4 for 4 days. *Bhlha15*$^{fl/fl}$ and *Xbp1*$^{fl/fl}$ mice served as controls. **(A)** Normal *in vitro* differentiation of PBs (CD23^{-}CD138^{+}) from splenic B cells of all three genotypes. One of five experiments is shown. **(B)** ELISPOT analysis of *in vitro* differentiated PBs. At day 4 of LPS plus IL-4 stimulation, 200 CD23^{-}CD138^{+}IgM^{+} plasmablasts were sorted and plated per well for 6 hours. Plates were coated and developed with anti-IgM. One of 2 independent experiments is shown. The scale bar indicates 2 mm. **(C)** Size distribution of the antibody-containing dots, which were produced by the IgM ASCs shown in (**B**). The dot sizes were automatically quantified by using the Fiji software (see Methods). Black lines indicate the median, and boxes represent the middle 50% of the data. Whiskers denote all values of the 1.5× interquartile range. Dots with a size of > 0.001 mm^2 are analyzed. **(D, E)** Analysis of PBs that were generated *in vitro* in the iGB system by stimulation of splenic CD43^{-} B cells on 40LB cells for 4 days with IL-4 followed by 4 days with IL-21 stimulation. **(D)** ELISPOT analysis of IgE-secreting PBs. IgE^{+}CD138^{+}CD19^{+}CD23^{-} PBs were sorted, and 200 cells were plated per well. It is important to note that most B cells have undergone class switch recombination to IgE after 8 days in the iGB system (data not shown). One of 2 experiment is shown. The scale bar indicates 2 mm. **(E)** Size distribution of the antibody-containing dots, which were produced by the IgE ASCs shown in (**D**) and were analyzed as described in (**C**). Statistical data (**A**-**E**) are indicated as mean values with SEM (**A**, **B**, **D**) and were analyzed with the unpaired Student's *t* test (**A**, **B**, **D**) or Mann-Whitney test (**C**, **E**); ***$P < 0.001$; ****, $P < 0.0001$. Each dot (**A**, **B**, **D**) represents one mouse.

SUPPLEMENTARY FIGURE 4 | Normal morphological structure of *Cd23*-Cre *Bhlha15*$^{fl/fl}$ plasma cells. **(A)** Electron microscopic analysis of bone marrow PCs of the indicated genotypes, which were sorted as B220^{int}CD28^{+}CD138^{+}Lin^{-} cells, fixed and processed as described in Methods. The scale bars indicate 2 µm. The cells surrounding the PCs are erythrocytes. **(B, C)** Golgi-Tracker (**B**) and ER-Tracker **(C)** staining of PCs at day 7 after NP-KLH immunization. A representative flow-cytometric analysis (left) and quantification of the MFI values (right) of one experiment are shown. Statistical data (**B**, **C**) are shown as mean values with SEM and were analyzed with the unpaired Student's *t* test; *$P < 0.05$; **$P < 0.01$; ****, $P < 0.0001$. Each dot (**B**, **C**) represents one mouse.

SUPPLEMENTARY FIGURE 5 | Mist1-dependent gene expression program in plasma cells.
(A) Immunomagnetic enrichment of $CD138^{+}$ PBs that were *in vitro* differentiated for 4 days by LPS stimulation of $CD43^{-}$ B cells from the spleen of *Bhlha15*$^{fl/fl}$ *Rosa26*$^{BirA/BirA}$ mice. The $CD138^{+}$ PBs were enriched with CD138-MicroBeads, and their purity was determined by flow cytometric analysis together with the input material and flowthrough fraction prior to Bio-ChIP-seq analysis. **(B)** Published consensus E2A motif identified in PBs (*5*). **(C)** Extensive overlap of the previously published E2A peaks, identified in PBs (*5*), with the Mist1 peaks defined in this study. **(D)** Streptavidin (SA) pulldown of E2A together with Mist1-Bio from a nuclear extract of *Bhlha15*$^{fl/fl}$ *Rosa26*$^{BirA/BirA}$ PBs after 4 days of LPS stimulation. The input material (1%), unbound fraction (0.5%) and precipitated proteins were analyzed by immunoblotting with an anti-E2A or anti-Mist1 antibody. It is important to note that the weak E2A signal may reflect a relatively weak interaction between Mist1-Bio and E2A, while the interaction between Mist1-Bio and the streptavidin-beads is very strong. As a consequence, all Mist1-Bio molecules are pulled down from the nuclear extract with streptavidin, while only the E2A protein faction bound to Mist1-Bio at the time of the pull-down are co-precipitated. **(E)** Principal component analysis based on 2 RNA-seq experiments performed with splenic PCs of each indicated genotype at day 7 after NP-KLH immunization. **(F**, **G)** Binding of Mist1 and E2A at the *Irf4* **(F)** and *Prdm1* **(G)** loci in LPS-stimulated PBs. The expression (RNA-seq data) in control and *Cd23*-Cre *Bhlha15*$^{fl/fl}$ (KO) PCs is shown together the Mist1 Bio-ChIP-seq data and the previously published E2A ChIP-seq and ATAC-seq data of LPS-differentiated PBs (*5*). Horizontal bars indicate peaks identified by MACS peak calling. The elements A-H upstream of the *Prdm1* gene were previously shown to interact with the *Prdm1* promoter by 3C-analysis (*5*). **(H)** Expression of 8 genes, which are activated more than 3-fold by Mist1 in PCs as well as by Xbp1 in PBs.

REFERENCES

1. J. Tellier *et al.*, Blimp-1 controls plasma cell function through the regulation of immunoglobulin secretion and the unfolded protein response. *Nat. Immunol.* **17**, 323-330 (2016).
2. A. Jolma *et al.*, DNA-binding specificities of human transcription factors. *Cell* **152**, 327-339 (2013).
3. R. Revilla-i-Domingo *et al.*, The B-cell identity factor Pax5 regulates distinct transcriptional programmes in early and late B lymphopoiesis. *EMBO J.* **31**, 3130-3146 (2012).
4. M. Minnich *et al.*, Multifunctional role of the transcription factor Blimp-1 in coordinating plasma cell differentiation. *Nat. Immunol.* **17**, 331-343 (2016).
5. M. Wöhner *et al.*, Molecular functions of the transcription factors E2A and E2-2 in controlling germinal center B cell and plasma cell development. *J. Exp. Med.* **213**, 1201-1221 (2016).
