# Supplementary material for: The Xbp1-regulated transcription factor Mist1 restricts antibody secretion by restraining Blimp1 expression in plasma cells
Source: Front Immunol. 2022 Dec 21;13:859598. doi: 10.3389/fimmu.2022.859598 (PMC9811352; doi:10.3389/fimmu.2022.859598)
Supplement: Supplementary file 5 [file DataSheet_2.pdf]

# Supplementary Materials and Methods

## Wöhner et al.

### Mice

The *Bhlha15*$^{fl/fl}$ mice (*1*), *Xbp1*$^{fl/fl}$ mice (*2*), Rosa26$^{BirA/BirA}$ mice (*3*), *Meox2*$^{Cre/+}$ mice (*4*), transgenic *Cd23-Cre* mice (*5*), *Prdm1*$^{Gfp/+}$ mice (*6*) and *Bhlha15*$^{CreER/+}$ mice (*7*) mice were maintained on the C57BL/6 genetic background. All animal experiments were carried out according to valid project licenses, which were approved and regularly controlled by the Austrian Veterinary Authorities.

### Generation of the *Xbp1s*$^{Bio}$ allele

The *Xbp1s*$^{Bio}$ allele (**Supplementary Figure 1E**) was generated by homologous recombination in mouse ES cells. The corresponding targeting vector was obtained by insertion of the following sequences in the 5′ to 3′ direction into the mouse *Xbp1* bacterial artificial chromosome (BAC) RP23-421C23 by recombination-mediated genetic engineering in *E. coli*; a 185-bp fragment containing the C-terminal tag sequences (fused in frame to the last Xbp1s codon) and a 1.9-kb fragment containing the human β-actin promoter linked to the neomycin-resistance gene (flanked by *lox*P sites). The tag sequences contained a TEV protease cleavage site, epitopes for anti-V5 and anti-Flag antibodies, a PreScission protease cleavage site and a biotin acceptor sequence (Bio) for biotinylation by the *E. coli* biotin ligase BirA. In a second step, the targeting vector was generated by excision and insertion of the integrated sequences together with the flanking 5′ (4.6 kb) and 3′ (1.9 kb) homology regions by recombination-mediated genetic engineering from the modified BAC into the pBV-DTA-pA plasmid containing a gene encoding the herpes simplex virus thymidine kinase (for negative selection). Linearized DNA (15 μg) was electroporated into cells ($1 \times 10^7$) of the hybrid C57BL/6 x 129Sv ES cell line A9, followed by selection with 250 μg/ml G418. PCR-positive clones were verified by Southern blot analysis prior to injection into C57BL/6 blastocysts and the generation of *Xbp1s*$^{Bio\text{-}neo/+}$ mice, which were subsequently crossed with *Meox2*$^{Cre/+}$ mice (*4*) to generate *Xbp1s*$^{Bio/+}$ mice, followed by backcrossing to the C57BL/6 genetic background.

### Antibodies and intracellular flow cytometry

The following monoclonal antibodies were used for flow cytometric analysis of the mouse spleen, bone marrow and cultured PBs: B220/CD45R (RA3-6B2), CD4 (GK1.5), CD8a (53-6.7), CD11b/Mac1 (M1/70), CD19 (1D3), CD21/CD35 (7G6), CD22 (Cy34.1), CD23 (B3B4), CD28 (37.51), CD95/Fas (Jo2), CD138 (281-2), IgD (11.26c), IgM (II/41), IgE (R35-72), IgG1 (A85-1), GL7 (GL-7), Xbp1 (M-186), Mist1 (D7N48), Blimp1 (5E7), TACI/CD267 (8F10), Irf4 (IRF4.3E4) antibodies. For labeling of the lysosomes, LysoTracker Deep Red (Thermo Fisher Scientific) staining was performed at 50 nM in medium for 30 min at 37 °C. Cells were washed twice and then stained with additional antibodies. For labelling of the ER and Golgi, cells were labelled with the Golgi/ER staining kit Cytopainter (Abcam) according to the manufacturer's instructions. For intracellular staining, we used the Foxp3 Staining Buffer Set (Miltenyi Biotec).

**Definition and flow cytometric sorting of cell populations**

Long-lived plasma cells (PCs) from the bone marrow, PCs and germinal center (GC) B cells from the spleen and *in vitro* differentiated activated cells, pre-plasmablasts (pre-PBs) and plasmablasts (PBs) were sorted with a FACS Aria machine (Becton Dickinson) as follows: recirculating B cells ($CD19^{+}B220^{+}IgD^{hi}IgM^{lo}$), GC B cells ($CD19^{+}B220^{+}CD93^{+}GL7^{+}$), PCs ($TACI^{+}CD138^{+}$ or Blimp1-$GFP^{+}CD138^{+}$), *in vitro* activated B cells ($CD19^{+}CD23^{+}CD138^{-}$), pre-PBs ($CD19^{+}CD23^{-}CD138^{-}$) and PBs ($CD19^{+}CD23^{-}CD138^{+}$).

***In vitro*** **B cell stimulation experiments**

Our LPS or LPS plus IL-4 stimulation procedures resulted in about 80% pre-PBs and PBs and 20% activated B cells after 4 days of stimulation. For this, $CD43^{-}$ splenic B cells were isolated by immunomagnetic sorting and seeded at a density of 5 x $10^5$ cells/ml in IMDM medium containing 10% fetal calf serum (Thermo Fisher), 1 mM glutamine, 50 µM β-mercaptoethanol and 25 µg/ml LPS (L4130 Sigma-Aldrich) or IL-4 (20 ng/ml) and 25 µg/ml LPS.

For experiments in the iGB system, 40LB cells were cultured in DMEM medium supplemented with 10% fetal calf serum prior to irradiation, and 1 x $10^5$ splenic B cells were plated on the 40LB cells in a well of a 6-well plate in RPMI medium supplemented with 10% fetal calf serum (Thermo Fisher Scientific), 1 mM glutamine, 50 µM β-mercaptoethanol, 10 mM Hepes and 1 mM sodium pyruvate. The cells were subsequently treated with IL-4 (20 ng/ml) for 4 days. At day 4, 1 x $10^5$ stimulated B cells were transferred onto a fresh 40LB cell layer in a well of a 6-well plate and were incubated in medium containing IL-21 (10 ng/ml) for the next 4 days.

**Immunization, ELISPOT and ELISA analyses**

The immune response to a T cell-dependent antigen was studied by intraperitoneal injection of 100 µg of 4-hydroxy-3-nitrophenylacetyl-conjugated keyhole limpet hemocyanin (NP-KLH) in alum. The frequencies of antibody-secreting cells (ASCs) were determined in the spleen and bone marrow by enzyme-linked immunospot (ELISPOT) assay as described (*8*). Anti-IgM, anti-IgG or $NP_{24}$-BSA coated plates were used for capturing antibodies secreted by individual cells, respectively. Spots were visualized with goat anti-mouse IgM, IgG or IgG1 antibodies conjugated to alkaline phosphatase (Southern Biotechnology Associates), and color was developed by the addition of BCIP/NBT Plus solution (Southern Biotechnology Associates). After extensive washing, the spots were counted with an ELISPOT Reader System (Autoimmun Diagnostika). The same procedure was used for *in vitro* cultured cells, for which the ELISPOT plated were coated with anti-mouse IgE or IgM antibodies.

The serum titer of NP-specific IgG1 and IgG2b antibodies was determined by enzyme-linked immunosorbent assay (ELISA) (*8*) by using plates, which were coated with 25 µg/ml $NP_{24}$-BSA to capture NP-specific IgG1 and IgG2b antibodies, respectively. The serum concentration of NP-specific IgG1 was determined relative to that of a standard anti-NP IgG1 antibody (hybridoma SSX2.1). For serum titers of unimmunized mice, a similar approach was used by coating the plates with anti-mouse IgM, IgG1, IgG2b, IgG3, IgA or IgE. Serial dilutions of the respective antibody isoforms at defined concentrations were plated and used as standards.

**Measurement of the ELISPOT size**

The size of all ELISPOTs in each well were analyzed using a customized macro developed in the Fiji macro language to perform automated segmentation and measurement in batch mode. Borders of the well area were defined to exclude outside reflections from the analysis, then color deconvolution was used to separate ELISPOTs from background. Laplace filtering and background subtraction were used to enhance contrast and to create binary images of the objects. Segmentation was performed in three rounds to cover different sizes, and the resulting images were then merged. The segmented images were then refined, and objects with low contrast were excluded. The resulting areas were measured.

**Calculation of ER mass**

EM pictures of PCs were analyzed using the Fiji software by manually marking the ER structures and adding up the resulting areas measured on the EM section.

**Glycosylation analysis**

For glycosylation analyses, approximately 1 µg of murine serum IgG was purified using protein G Dynabeads (Thermo Fisher Scientific) and separated by SDS-PAGE. The Coomassie-stained SDS-PAGE bands representing the IgG heavy-chain polypeptides were excised, destained, reduced, alkylated and in-gel digested with trypsin (Trypsin Gold sequencing grade, Promega) as described previously (*9, 10*). Tryptic peptides and glycopeptides were separated by reversed-phase nano-HPLC (Acclaim PepMap C18, 75 µm x 50 cm, Thermo Fisher Scientific) and analyzed by ESI-MS/MS (Q Exactive Hybrid Quadrupole-Orbitrap, Thermo Fisher Scientific). The relative abundances of IgG-subclass-specific glycopeptide glycoforms were calculated by integration (FreeStyle 1.3, Thermo Fisher Scientific) of the respective extracted ion chromatographs.

**Electron microscopy**

PC sorting was performed with 8 *Xbp1*$^{fl/fl}$, 4 *Cd23*-Cre *Xbp1*$^{fl/fl}$ and 3 *Cd23*-Cre *Bhlha15*$^{fl/fl}$ mice. PCs from the bone marrow of these mice were isolated by erythrocyte lysis, followed by MACS enrichment and flow cytometric sorting. For this, the cells were stained with biotinylated anti-CD4, anti-CD8 and anti-CD21 antibodies, followed by incubation with anti-biotin, anti-CD11b and anti-B220 MicroBeads (Miltenyi) and separation on a magnetic column. The non-bound cells in the flowthrough were stained for flow-cytometric sorting of Lin$^{-}$B220$^{int}$CD138$^{hi}$CD28$^{+}$ PCs. Cells were sorted in medium and fixed for 1 h in pre-warmed 2.5% glutaraldehyde in 0.1 M sodium phosphate buffer, pH 7.4. The solution was then centrifuged at 500 rcf for 3 min. Due to the small pellet size, the supernatant was carefully aspirated, and the pelleted cells were mixed with prefixed erythrocytes to minimize cell loss. The samples were then rinsed with the same buffer, post-fixed in 2% osmium tetroxide in 0.1 M sodium phosphate buffer, pH 7.4, on ice, dehydrated in a graded series of acetone on ice and embedded in Agar 100 resin. 70-nm sections were cut parallel to the substrate and post-stained with 2% uranyl acetate and Reynolds lead citrate. Sections were examined with a FEI Morgagni 268D (FEI, Eindhoven, The Netherlands) operated at 80 kV. Images were acquired using an 11-megapixel Morada CCD camera (Olympus-SIS).

**cDNA preparation for RNA-sequencing**

For RNA-seq analysis of Xbp1-regulated genes, splenic B cells were cultured for 8 days in the iGB system, as described above, and were sorted as $CD19^{+}CD138^{+}CD23^{-}$ PBs on day 8. For RNA-seq analysis of Mist1-regulated genes, mice were immunized with NP-KLH (in alum), and, at day 7, the splenic PCs were sorted as $TACI^{+}CD138^{+}$ cells. Total RNA was prepared with the Monarch Total RNA Miniprep Kit T2010S (NEB), followed by mRNA and cDNA preparation using the NEBNext Ultra II Directional RNA Kit Prep Kit for Illumina.

**Bio-ChIP analysis**

$CD43^{-}$ B cells from the spleen of *Bhlha15*$^{fl/fl}$ *Rosa26*$^{BirA/BirA}$ PBs or *Xbp1*$^{Bio/Bio}$ *Rosa26*$^{BirA/+}$ mice were stimulated for 4 days with LPS. For Mist1 Bio-ChIP analysis, $CD138^{+}$ PBs were stained with CD138-PE (Miltenyi) followed by immunomagnetic sorting with CD138-MicroBeads (Miltenyi) and crosslinking at room temperature for 10 min with 1% formaldehyde. For Xbp1 Bio-ChIP analysis, the differentiated cells at day 4 of LPS stimulation were directly used for crosslinking with 1% formaldehyde for 10 min. Nuclei were prepared and lyzed, and the chromatin was sheared with a Bioruptor® sonicator (Diagenode) followed by immunoprecipitation using the streptavidin magnetic beads (Dynabeads M-270, Thermo Fisher) as described (*11*).

**Library preparation and Illumina deep sequencing**

About 1-5 ng of cDNA or ChIP-precipitated DNA were used as starting material for the generation of sequencing libraries with the NEBNext Ultra II DNA Library Prep Kit for Illumina. Cluster generation and sequencing was carried out by using the Illumina HiSeq 2000 system with a read length of 50 nucleotides according to the manufacturer's guidelines.

**Bioinformatic analysis of ChIP-seq data**

Sequence reads that passed the Illumina quality filtering were considered for alignment. For ChIP-seq, the reads were aligned to the mouse genome assembly version of July 2007 (NCBI37/mm9), using the Bowtie program (*12*) version 1.0.0 for experimental samples and version 0.12.5. for the input sample. Read coverage tracks were calculated with the BEDTool program version 2.27.1 (*13*), were normalized to reads per millions (RPM) using the SAMTools version 1.9 (*14*) as well as the KentTools version 20190507 (*15*). Where necessary (e.g. for comparison of peak regions), reads were down-sampled before peak calling to the lowest read number in the samples to be compared, using the Picard tool version 2.18.27 (*16*). Peaks were called with the MACS program version 2.2.5 (*17*), using a mature B cell input sample (GSM2058441) as a control. We filtered all peaks for significance ($P$ value < $10^{-10}$). Peak overlaps were calculated with the MULTOVL program version 1.3 (*18*). All analyses were implemented as bash and R version 3.6 scripts.

**Bioinformatic analysis of RNA-seq data**

Sequence reads that passed the Illumina quality filtering were considered for alignment. The reads were adapter-trimmed with cutadapt and aligned with STAR version 2.4.2. to the mouse genome assembly version of July 2007 (NCBI37/mm9). Read counting was done with FeatureCounts version 1.5 and converted to TPMs using R 3.6 and custom scripts. Normalization

and differential analysis was performed with the DESeq2 version 1.24.0 (*19*). For RNA-seq comparison of the Xbp1-dependent gene expression in PBs, we considered the samples 124150-124155 (GSM5823267- GSM5823272; **Supplementary Table 3**) together for normalization and dispersion estimation. For RNA-seq comparison of the Mist1-dependent gene expression in splenic PCs, we used samples 137948-137951 (GSM5726098-GSM5726101; **Supplementary Table 3**) together for normalization and dispersion estimation. The data of the experimental genotypes were finally compared with those of the controls, and genes with an expression difference of > 3-fold, an adjusted *P* value of < 0.05 (Wald-test), an expression value of > 5 TPM (at least in one sample) were considered significant. The immunoglobulin and T cell receptor genes were excluded from the TPM calculation. Gene set enrichment analysis (GSEA) was performed using the GSEA software from the Broad Institute (*20*). Genes were ranked based on their shrunken $\log_2$-fold change, determined by the DESeq2 package, and were compared to the gene sets described in the manuscript. All analyses were implemented as R version 3.6 scripts.

**Statistical analysis**

Statistical analysis was performed with the GraphPad Prism 8 software. Unpaired Student's *t*-tests were used to assess the statistical significance of one observed parameter between two experimental groups. The Mann-Whitney test was used to assess the statistical significance of the size of a multitude of ELISPOTs between three experimental groups.

**Data availability**

The RNA-seq and ChIP-seq data (**Supplementary Table 3**), which were generated for this study, are available at the Gene Expression Omnibus (GEO) repository under the accession number GSE190591.

**References**


1. A. Karki *et al.*, Silencing Mist1 gene expression is essential for recovery from acute pancreatitis. *PLoS One* **10**, e0145724 (2015).
2. C. Hetz *et al.*, Unfolded protein response transcription factor XBP-1 does not influence prion replication or pathogenesis. *Proc. Natl. Acad. Sci. USA* **105**, 757-762 (2008).
3. S. Driegen *et al.*, A generic tool for biotinylation of tagged proteins in transgenic mice. *Transgenic Res* **14**, 477-482 (2005).
4. M. D. Tallquist, P. Soriano, Epiblast-restricted Cre expression in MORE mice: a tool to distinguish embryonic vs. extra-embryonic gene function. *Genesis* **26**, 113-115 (2000).
5. K. Kwon *et al.*, Instructive role of the transcription factor E2A in early B lymphopoiesis and germinal center B cell development. *Immunity* **28**, 751-762 (2008).
6. A. Kallies *et al.*, Plasma cell ontogeny defined by quantitative changes in Blimp-1 expression. *J. Exp. Med.* **200**, 967-977 (2004).
7. G. Shi *et al.*, Loss of the acinar-restricted transcription factor Mist1 accelerates Kras-induced pancreatic intraepithelial neoplasia. *Gastroenterology* **136**, 1368-1378 (2009).
8. K. G. Smith, A. Light, G. J. Nossal, D. M. Tarlinton, The extent of affinity maturation differs between the memory and antibody-forming cell compartments in the primary immune response. *EMBO J.* **16**, 2996-3006 (1997).

9. J. Stadlmann, M. Pabst, D. Kolarich, R. Kunert, F. Altmann, Analysis of immunoglobulin glycosylation by LC-ESI-MS of glycopeptides and oligosaccharides. *Proteomics* **8**, 2858-2871 (2008).
10. D. Maresch, F. Altmann, Isotype-specific glycosylation analysis of mouse IgG by LC-MS. *Proteomics* **16**, 1321-1330 (2016).
11. R. Revilla-i-Domingo *et al.*, The B-cell identity factor Pax5 regulates distinct transcriptional programmes in early and late B lymphopoiesis. *EMBO J.* **31**, 3130-3146 (2012).
12. B. Langmead, C. Trapnell, M. Pop, S. L. Salzberg, Ultrafast and memory-efficient alignment of short DNA sequences to the human genome. *Genome Biol.* **10**, R25 (2009).
13. A. R. Quinlan, I. M. Hall, BEDTools: a flexible suite of utilities for comparing genomic features. *Bioinformatics* **26**, 841-842 (2010).
14. H. Li *et al.*, The Sequence Alignment/Map format and SAMtools. *Bioinformatics* **25**, 2078-2079 (2009).
15. R. M. Kuhn, D. Haussler, W. J. Kent, The UCSC genome browser and associated tools. *Brief. Bioinform.* **14**, 144-161 (2013).
16. A. McKenna *et al.*, The Genome Analysis Toolkit: a MapReduce framework for analyzing next-generation DNA sequencing data. *Genome Res.* **20**, 1297-1303 (2010).
17. Y. Zhang *et al.*, Model-based analysis of ChIP-Seq (MACS). *Genome Biol.* **9**, R137 (2008).
18. A. Aszódi, MULTOVL: fast multiple overlaps of genomic regions. *Bioinformatics* **28**, 3318-3319 (2012).
19. M. I. Love, W. Huber, S. Anders, Moderated estimation of fold change and dispersion for RNA-seq data with DESeq2. *Genome Biol.* **15**, 550 (2014).
20. A. Subramanian *et al.*, Gene set enrichment analysis: a knowledge-based approach for interpreting genome-wide expression profiles. *Proc. Natl. Acad. Sci. USA* **102**, 15545-15550 (2005).
