# Supplementary figures and images for: The Xbp1-regulated transcription factor Mist1 restricts antibody secretion by restraining Blimp1 expression in plasma cells
Source: Front Immunol. 2022 Dec 21;13:859598. doi: 10.3389/fimmu.2022.859598 (PMC9811352; doi:10.3389/fimmu.2022.859598)

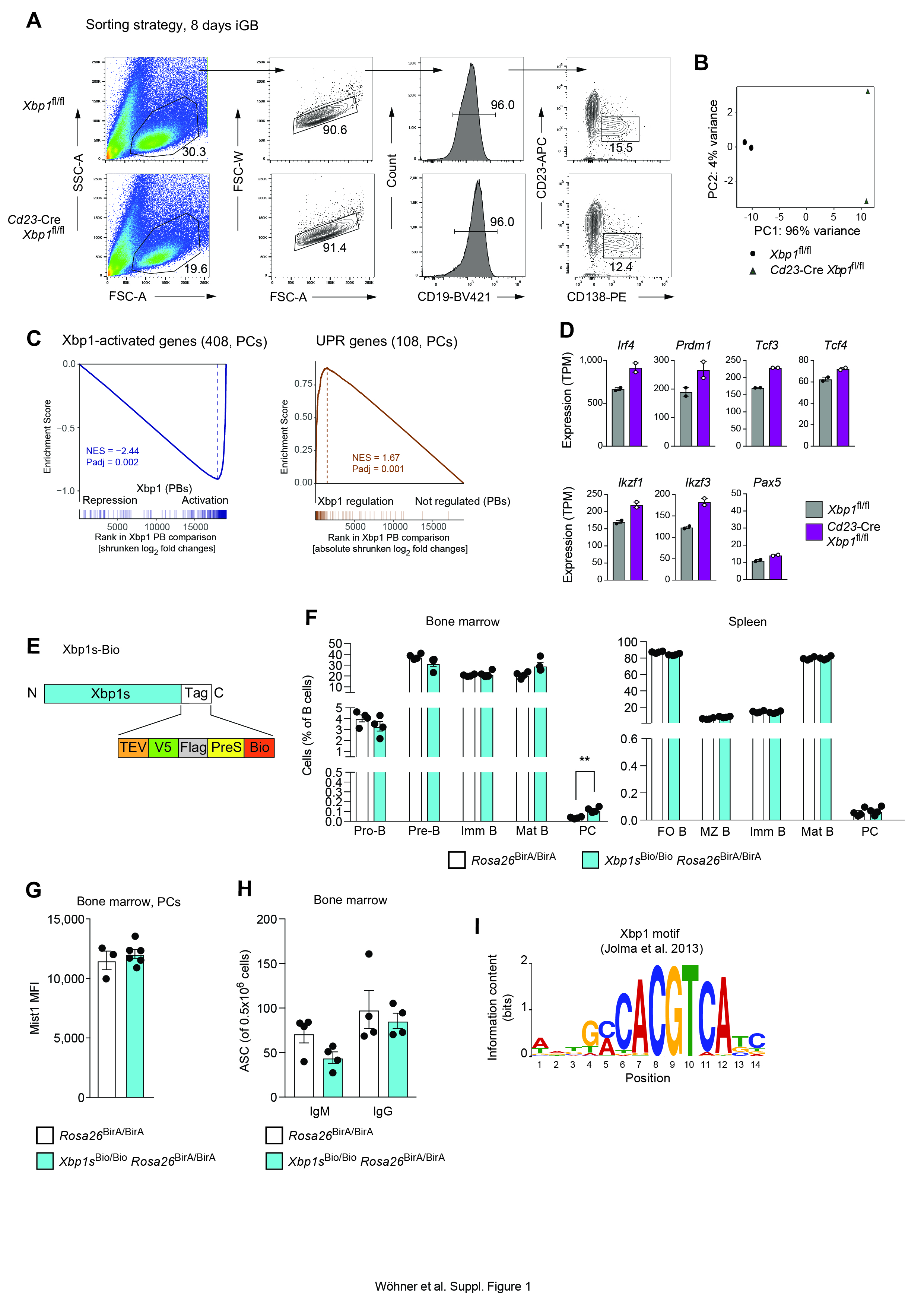

Supplement: Supplementary file 6 [file Image_1.jpeg]

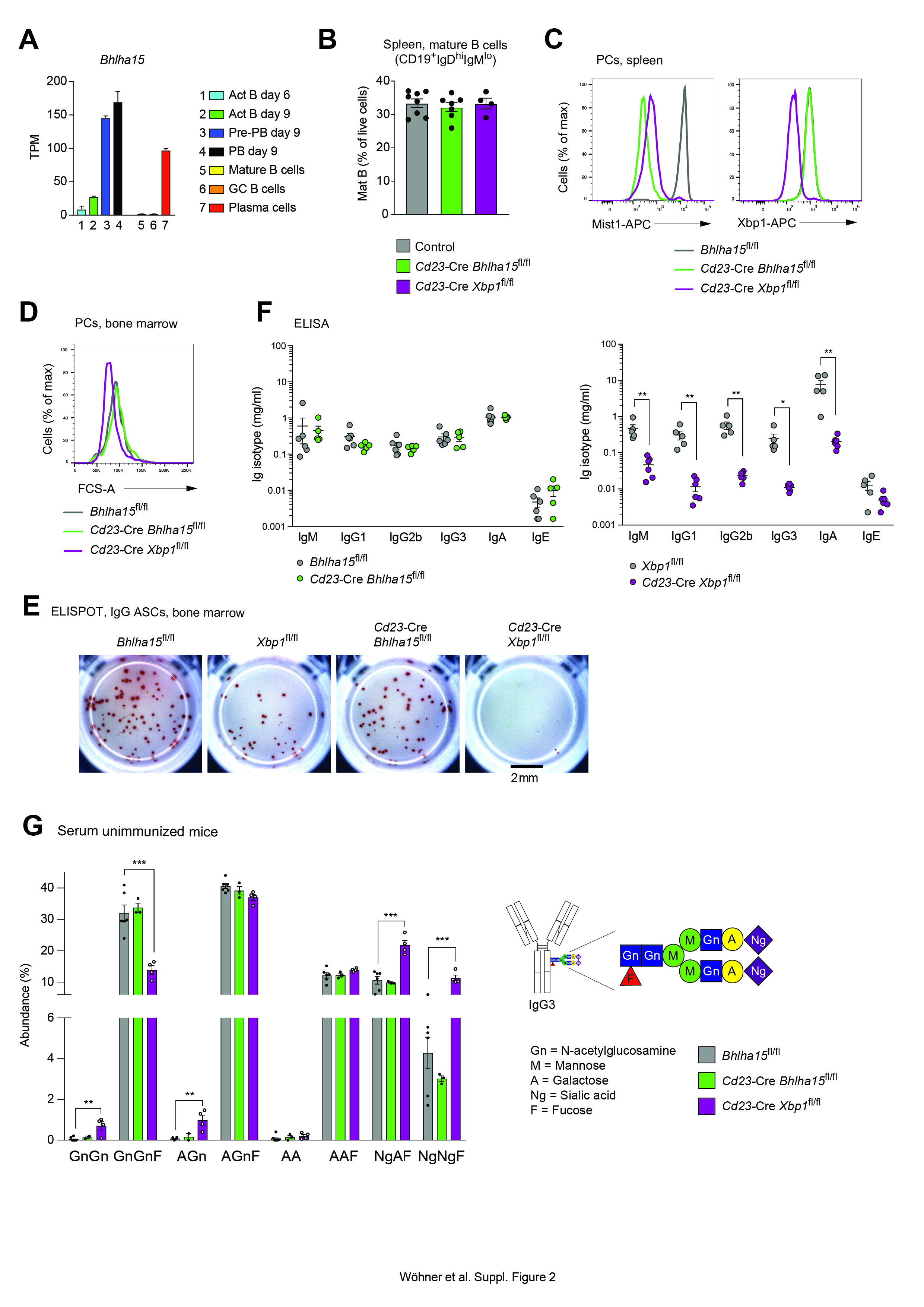

Supplement: Supplementary file 7 [file Image_2.jpeg]

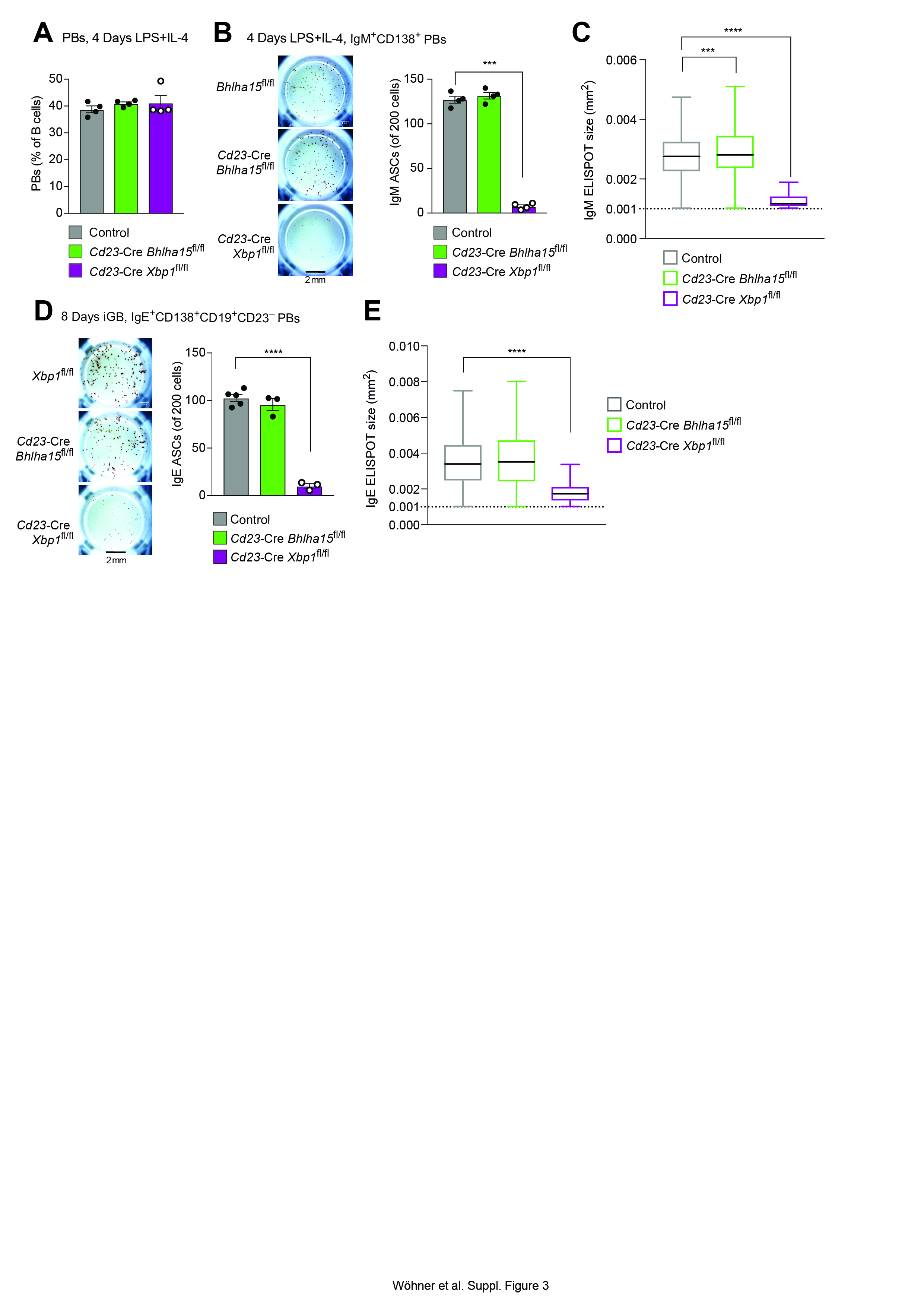

Supplement: Supplementary file 8 [file Image_3.jpeg]

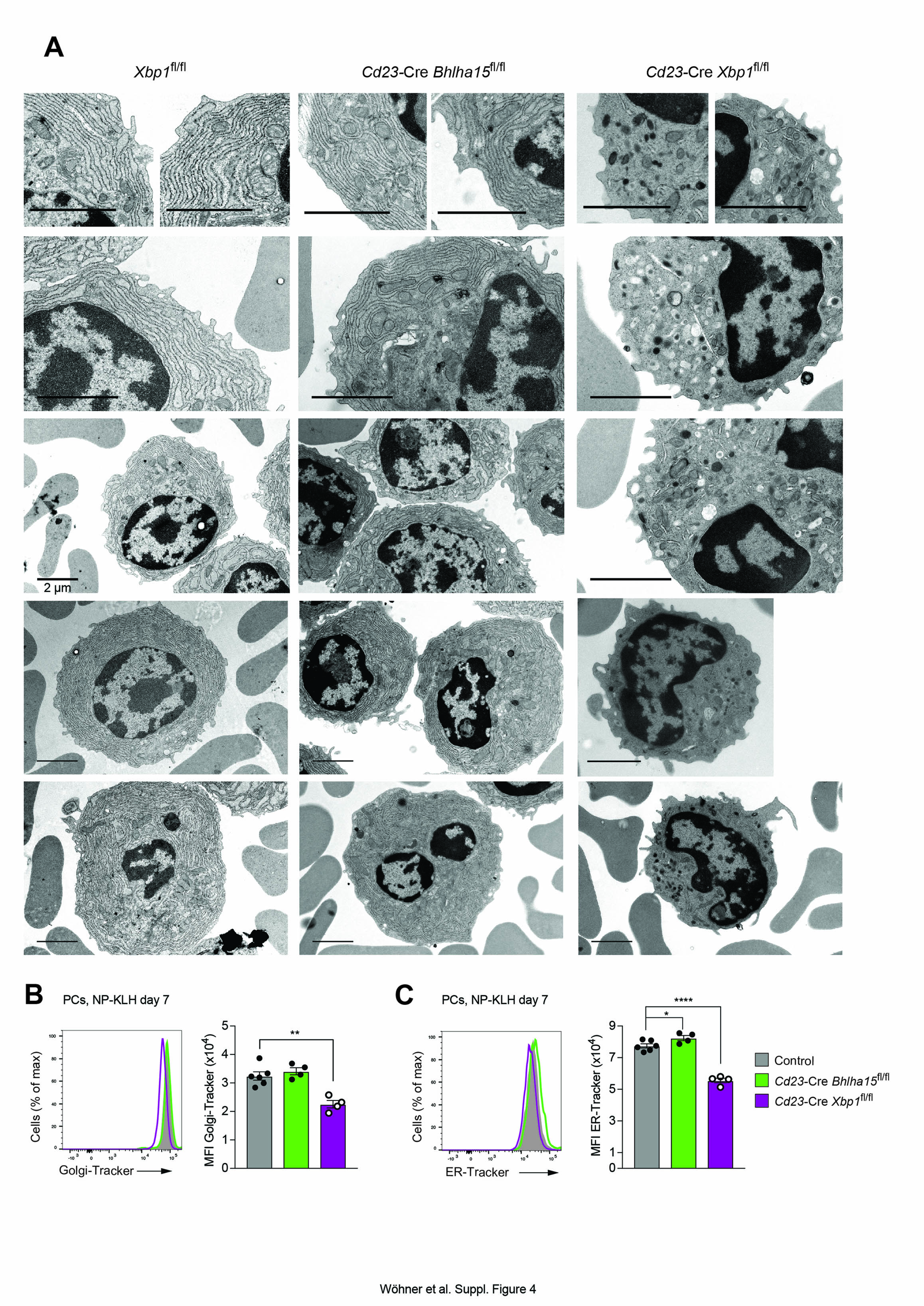

Supplement: Supplementary file 9 [file Image_4.jpeg]

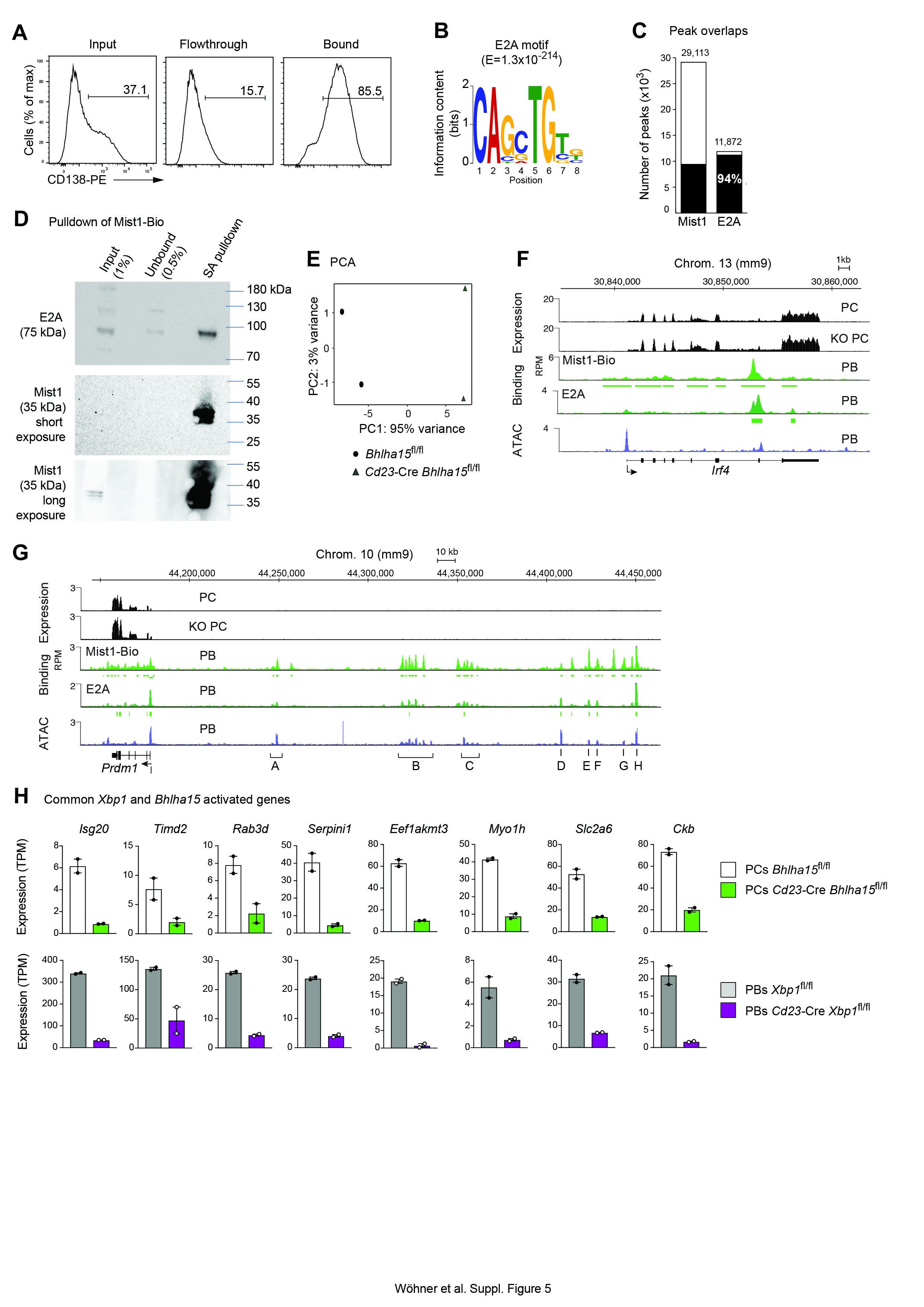

Supplement: Supplementary file 10 [file Image_5.jpeg]
